# Cyclometalated iridium-coumarin ratiometric oxygen sensors: improved signal resolution and tunable dynamic ranges†

**DOI:** 10.1039/d2sc02909j

**Published:** 2022-07-15

**Authors:** Yanyu Wu, Gregory D. Sutton, Michael D. S. Halamicek, Xinxin Xing, Jiming Bao, Thomas S. Teets

**Affiliations:** University of Houston, Department of Chemistry 3585 Cullen Blvd., Room 112 Houston TX 77204-5003 USA tteets@uh.edu; University of Houston, Department of Electrical and Computer Engineering and Texas Center for Superconductivity (TcSUH) Houston TX 77204 USA

## Abstract

In this work we introduce a new series of ratiometric oxygen sensors based on phosphorescent cyclometalated iridium centers partnered with organic coumarin fluorophores. Three different cyclometalating ligands and two different pyridyl-containing coumarin types were used to prepare six target complexes with tunable excited-state energies. Three of the complexes display dual emission, with fluorescence arising from the coumarin ligand, and phosphorescence from either the cyclometalated iridium center or the coumarin. These dual-emitting complexes function as ratiometric oxygen sensors, with the phosphorescence quenched under O_2_ while fluorescence is unaffected. The use of blue-fluorescent coumarins results in good signal resolution between fluorescence and phosphorescence. Moreover, the sensitivity and dynamic range, measured with Stern–Volmer analysis, can be tuned two orders of magnitude by virtue of our ability to synthetically control the triplet excited-state ordering. The complex with cyclometalated iridium ^3^MLCT phosphorescence operates under hyperoxic conditions, whereas the two complexes with coumarin-centered phosphorescence are sensitive to very low levels of O_2_ and function as hypoxic sensors.

## Abstract 2

Cyclometalated iridium(iii) coumarin complexes with improved signal resolution for ratiometric oxygen sensing are described. Dynamic ranges are tunable over >2 orders of magnitude.

## Graphical abstract

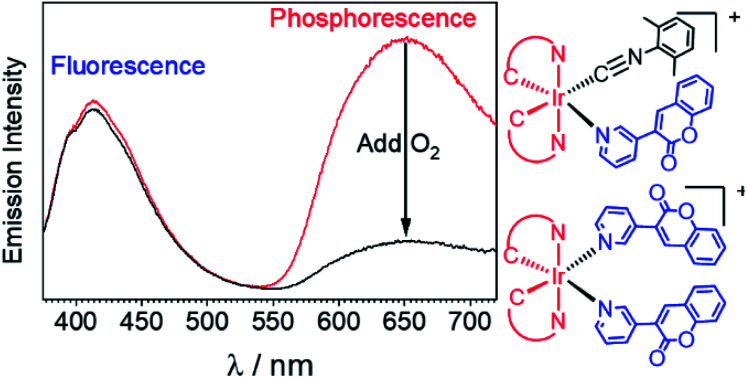

## Introduction

Chemical and biochemical mechanisms of aerobic metabolism largely depend on molecular oxygen.^1–3^ Tumor hypoxia is associated with a variety of common diseases^4^ and therefore the sensing of triplet oxygen has been a recent area of interest.^5–10^ The level of molecular oxygen in tumors is a good indicator of metabolic state and can help guide therapy for cancer treatment. Beyond applications in pathology, accurate oxygen sensing with good spatial and temporal resolution is critical for monitoring physiological responses to extreme environments, *e.g.* subsea, arctic, and outer space.^11^ Luminescent sensing of oxygen is particularly appealing, where the oxygen concentration is analyzed *via* the color, intensity, and/or lifetime of photoluminescence. In principle any phosphorescent compound can serve as an oxygen sensor, since molecular triplet states are efficiently quenched by O_2_.^12^ However, phosphorescent compounds give a “turn-off” response to oxygen, so their use requires accurate measurement of absolute luminescence intensity, which is prone to issues with calibration and reproducibility, resulting from variable excitation power, optical path length, and in biological applications heterogeneous cellular environments.^13^ Measurement of the phosphorescence lifetime attenuation works well in principle for sensing oxygen but is more technically complex. Luminescent ratiometric oxygen sensors avoid these limitations by incorporating two emission signals which are differentially modulated by O_2_, and hence the ratio of the two emission intensities can provide a simple and accurate readout of oxygen concentration.

Classical ratiometric oxygen sensors combine a fluorescent moiety with a molecular phosphor. These constructs have quenched phosphorescence in the presence of O_2_ while fluorescence is unaffected. Previous designs of ratiometric O_2_ sensors include quantum dots with phosphorescent metal complexes tethered to the surface^14^ and metal–organic frameworks (MOFs) that combine fluorescent and phosphorescent components.^15,16^ Polymer nanocomposites are also quite common as ratiometric oxygen sensors; these include polymer beads embedded with both molecular phosphors and fluorophores,^17–20^ fluorescent polymers with covalently attached or physically blended phosphorescent molecules,^21,22^ TADF copolymers where the ratio of prompt to delayed fluorescence is used to measure oxygen,^23^ and a supramolecular polymer-based palladium porphyrin nanoprobe which uses hydrogen bonding to link a phosphorescent indicator to a fluorescent reference dye.^24^ There are also single-component compounds with dual emission and ratiometric oxygen response, such as bimetallic lanthanide complexes from metal-centered excited states^25^ and boron clusters^26^ or lutetium porphyrin compounds^27^ with dual fluorescence/phosphorescence emission.

Among potential phosphorescent molecules for ratiometric sensing applications, cyclometalated iridium complexes, which have dominated the electroluminescence field,^28,29^ have perhaps the most desirable attributes. They are chemically robust, can be engineered to luminesce in any part of the visible or near-infrared spectrum, often with high quantum yields, and their phosphorescence lifetimes (on the order of microseconds) are well-suited for detecting typical atmospheric or physiological concentrations of O_2_. Nevertheless, only a few previously reported ratiometric oxygen sensors included cyclometalated iridium complexes. Dual-phosphorescent cyclometalated iridium complexes have been used for sensing hypoxia and hyperoxia.^30^ Heteroleptic cyclometalated iridium complexes with substituted bipyridine ligands have been synthesized to give dual emission in which the fluorescence occurs from the ^1^IL state located on the bipyridine and the phosphorescence arising from mixed ^3^IL and ^3^MLCT transitions.^31^ Cyclometalated iridium complexes have been linked to conjugated polymers which then function as ratiometric oxygen sensors,^32^ and most relevant to the present work, red-phosphorescent cyclometalated iridium complexes have been paired with blue-fluorescent coumarins to access biocompatible ratiometric O_2_ sensors.^5,33^ This latter design is effective, although the synthetic strategy involves upwards of 12 steps, owing primarily to the complex polyproline-substituted acetylacetonate spacer between the iridium center and the coumarin.

One of our primary goals in this area of research has been to design simpler, more generalizable synthetic strategies for preparing ratiometric oxygen sensors featuring cyclometalated iridium. In doing so, it would be much easier to modify the sensor attributes and optimize them for a specific application. The spectral profile, the resolution between the phosphorescence and fluorescence signals, the photoluminescence quantum yield, and the triplet lifetime, which are the critical determinants of the oxygen sensing dynamic range, can all in principle be quickly modified. Our initial foray into this area presented a series of bis-cyclometalated iridium complexes joined with pyridyl-substituted BODIPY fluorophores.^34^ The complexes are prepared in a few simple synthetic steps, the last one involving the generation of a substitutionally labile cyclometalated iridium intermediate that “snaps” together with the pyridyl-substituted BODIPY under mild conditions. This same synthetic strategy has been used in our group to access other multi-component photoactive structures, which are not ratiometric sensors but nonetheless are valuable platforms for studying excited-state energy transfer pathways.^35,36^ Some of the Ir-BODIPY compounds prepared in this way functioned as effective ratiometric oxygen sensors, with dynamic ranges that spanned hypoxic levels of O_2_ (*p*O_2_ ≤ 150 mm Hg).^34^

In this work, we present two significant advances in the design of ratiometric oxygen sensors featuring cyclometalated iridium, showing that the simple synthetic approach we have developed facilitates modification and optimization of the sensor attributes. One limitation of our first-generation Ir-BODIPY oxygen sensors is a significant overlap between the BODIPY's green fluorescence and the broad red phosphorescence from the iridium center. This poor signal resolution between the two luminescence channels means the effective ratiometric response never reaches zero, limiting the dynamic range. Here we study complexes that replace the green-fluorescent BODIPY with a blue-fluorescent coumarin, and in one such complex we also shift the phosphorescence deeper to the red, achieving greatly improved signal resolution showing no overlap between fluorescence and phosphorescence. While it is true that the blue fluorescence is not ideally suited for *in vivo* imaging, it does allow for greater wavelength separation between fluorescence and phosphorescence, which contributes to a much larger dynamic range for oxygen sensing that would benefit any potential application of these sensors. A second major discovery in this work, enabled by the ability to quickly “mix and match” organic fluorophores and bis-cyclometalated iridium phosphors, is the ability to tune the oxygen sensitivity over a wide range. We accomplish this by synthetic re-ordering of the triplet excited states. In some cases, the lowest triplet state is the typical metal-to-ligand charge transfer (MLCT) state common to most cyclometalated iridium complexes, which is sensitive to hyperoxic conditions. In other cases, a coumarin-centered triplet state is the T_1_ state, which is sensitive to hypoxic conditions. This type of synthetic control over relative triplet energies was previously used in dual-phosphorescent cyclometalated iridium sensors,^30^ and here we show it is also an effective approach for conventional fluorescent-phosphorescent dual-emissive probes. Two of our complexes have significant responses to oxygen levels below 50 mm Hg, which is critical for development of photodynamic therapy agents and for monitoring biological functions such as cellular ATP synthesis and gene amplification.^4^ Since different applications have very disparate requirements for oxygen-sensing response, the ability to synthetically tune the oxygen sensing range is paramount for continued development in this area. In total, this paper describes the preparation and characterization of six new cyclometalated iridium-coumarin complexes, and the three that show dual luminescence are subjected to an in-depth quantification of their oxygen-sensing attributes in abiological solutions.

## Results and discussion

### Synthesis of Ir-coumarin complexes

**Scheme 1:**
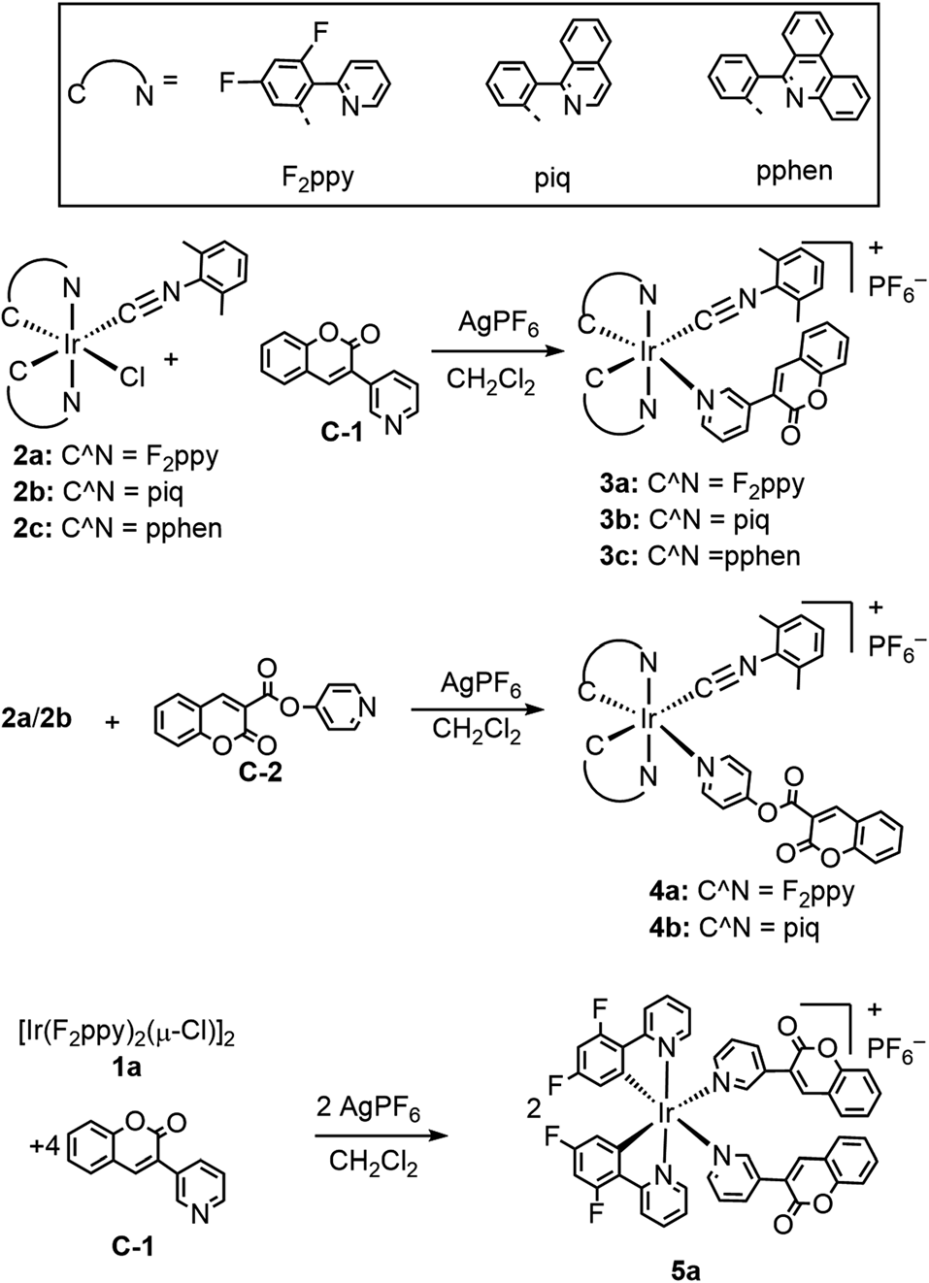
Synthesis of cyclometalated iridium-coumarin compounds.

The general synthetic preparation of the complexes is outlined in Scheme 1, and in most cases follows closely with the procedure used to prepare our first-generation Ir-BODIPY sensors.^34^ Three precursor classes were used to prepare the six compounds in this study. Two pyridyl-substituted coumarin compounds, abbreviated C-1 and C-2, were synthesized using known procedures.^37,38^ Chloro-bridged cyclometalated iridium dimers [Ir(C^N)_2_(μ-Cl)]_2_ (1a–c, C^N = cyclometalating ligand) are ubiquitous precursors accessed by the method of Nonoyama.^39^ In all iridium complexes described here, the letter in the numerical abbreviation denotes the cyclometalating ligand used, 2-(2,4-difluorophenyl)pyridine (F_2_ppy, a), 1-phenylisoquinoline (piq, b), and 6-phenylphenanthridine (pphen, c), which normally produce blue, red, and deep red phosphorescence when chelated to iridium(iii), respectively. The bis-cyclometalated iridium precursors 2a–c of the type Ir(C^N)_2_(CNAr^dmp^)(Cl) (CNAr^dmp^ = 2,6-dimethylphenylisocyanide) were synthesized as previously described by our group, cleaving the chloro-bridged dimers with isocyanides.^35,40^ Finally, the cyclometalated iridium-coumarin dyads 3a–c and 4a–b were prepared using simple, one-pot reactions between the respective coumarin and the isocyanide precursors 2a–c in the presence of AgPF_6_. Here we also introduce a new structural class where the bis-cyclometalated iridium center is coordinated to two pyridyl-coumarins, represented by complex 5a. This latter compound is prepared directly from the chloro-bridged cyclometalated iridium dimer 1a and coumarin C-1, also in the presence of AgPF_6_. These reactions are reasonably high-yielding based on crude NMR spectra but following multiple rounds of rigorous purification low to moderate isolated yields (20–60%) were obtained. ^1^H, ^19^F, and ^13^C{^1^H} NMR spectroscopy was performed on all the target complexes to affirm their identity and bulk purity. ^19^F NMR spectra were particularly useful for determining the *C*_1_ point group symmetry of 3a and 4a and the *C*_2_ symmetry of 5a; the former show four distinct ^19^F resonances for the F_2_ppy ligands, whereas the latter only shows two. The NMR spectra of the complexes can be found in Fig. S1–18 in the ESI.† To further confirm the identity of the target complexes, HRMS was performed on each compound and the results are shown in Fig. S19–24 of the ESI.† In four of the compounds [M–PF_6_]^+^ molecular ion peaks were resolved, whereas in 3c and 4b we observed dissociation of the pyridyl coumarin during ionization, with the corresponding peaks for the respective [Ir(C^N)_2_(CNAr^dmp^)]^+^ fragment observed instead.

**Fig. 1:**
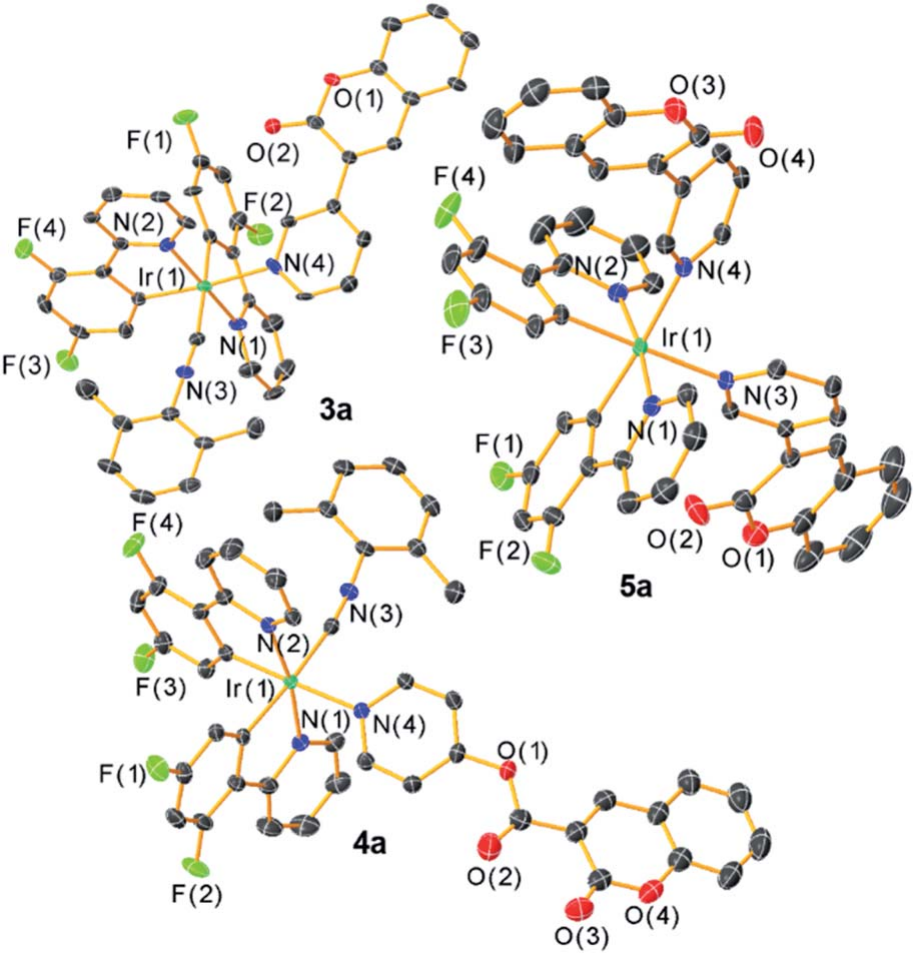
Molecular structures of complexes 3a–5a, determined by single-crystal X-ray diffraction. Ellipsoids are drawn at the 50% probability level with hydrogen atoms, solvent molecules and counterions omitted.

Single-crystal X-ray diffraction§§3a·0.5C_7_H_8_: CCDC 2126601, C_48.50_H_34_F_10_IrN_4_O_2_P, *M* = 1117.96, Triclinic, *P*-1, *a* = 12.373(6) Å, *b* = 19.541(9) Å, *c* = 21.137(10) Å, *α* = 63.579(5)°, *β* = 75.787(5)°, *γ* = 71.958(5)°, *Z* = 4, 16 458 tot. refln., 16 458 ind. refln., *R*_int_ = 0.0625, *R*_1_ = 0.045, *wR*_2_ = 0.242. 4a·1.5C_6_H_6_: CCDC 2126602, C_55_H_39_F_10_IrN_4_O_4_P, *M* = 1233.07, Triclinic, *P*-1, *a* = 12.0293(3) Å, *b* = 13.6426(3) Å, *c* = 15.8219(3) Å, *α* = 79.160(1)°, *β* = 81.948(1)°, *γ* = 71.953 (1), *Z* = 2, 31 358 tot. refln., 9473 ind. refln., *R*_int_ = 0.020, *R*_1_ = 0.023, *wR*_2_ = 0.058. 5a: CCDC 2126603, C_50_H_30_F_10_IrN_4_O_4_P, *M* = 1163.95, Triclinic, *P*-1, *a* = 11.885(2) Å, *b* = 13.195(3) Å, *c* = 16.517(3) Å, *α* = 105.127(2)°, *β* = 97.590(2)°, *γ* = 101.776(2), *Z* = 2, 33 955 tot. refln., 11 009 ind. refln., *R*_int_ = 0.032, *R*_1_ = 0.030, *wR*_2_ = 0.075. further confirms the molecular structures of the three F_2_ppy complexes 3a–5a, which are shown in Fig. 1. Refinement and diffraction data for these three complexes are summarized in Table S1 of the ESI.† All the complexes show a distorted octahedral geometry centered on the Ir atom. The pyridyl nitrogen atoms from the F_2_ppy ligands are in a trans orientation, with the other two *cis*-oriented coordination sites are occupied by one CNAr^dmp^ and one pyridyl coumarin ligand for complexes 3a and 4a, and two pyridyl coumarins in 5a. The *C*_1_ symmetry of 3a and 4a and the approximate *C*_2_ symmetry of 5a, readily apparent from their NMR spectra, are confirmed by the crystal structures. The Ir center is covalently bound to the coumarin *via* the N atom in the pyridyl moiety, with Ir–N bond distances ranging between 2.172 and 2.190 Å, slightly longer than the Ir–N bond distances between the F_2_ppy nitrogen atom and the iridium center, which range from 2.046 to 2.079 Å.

### Photophysical properties

Fig. S25 and S26 of the ESI† display the overlaid UV-vis absorption and photoluminescence emission spectra of free coumarins C-1 and C-2. The absorption spectra of both coumarins show two near-UV absorption peaks between 300–350 nm, which can be assigned to S_0_→S_1_ and S_0_→S_2_ transitions. Both C-1 and C-2 display deep-blue fluorescence emission with maxima at *λ* = 406 nm and 420 nm, respectively, with moderate Stokes shifts of 81 nm (C-1) and 86 nm (C-2), corresponding to 6100 cm^−1^ for both compounds.

**Fig. 2:**
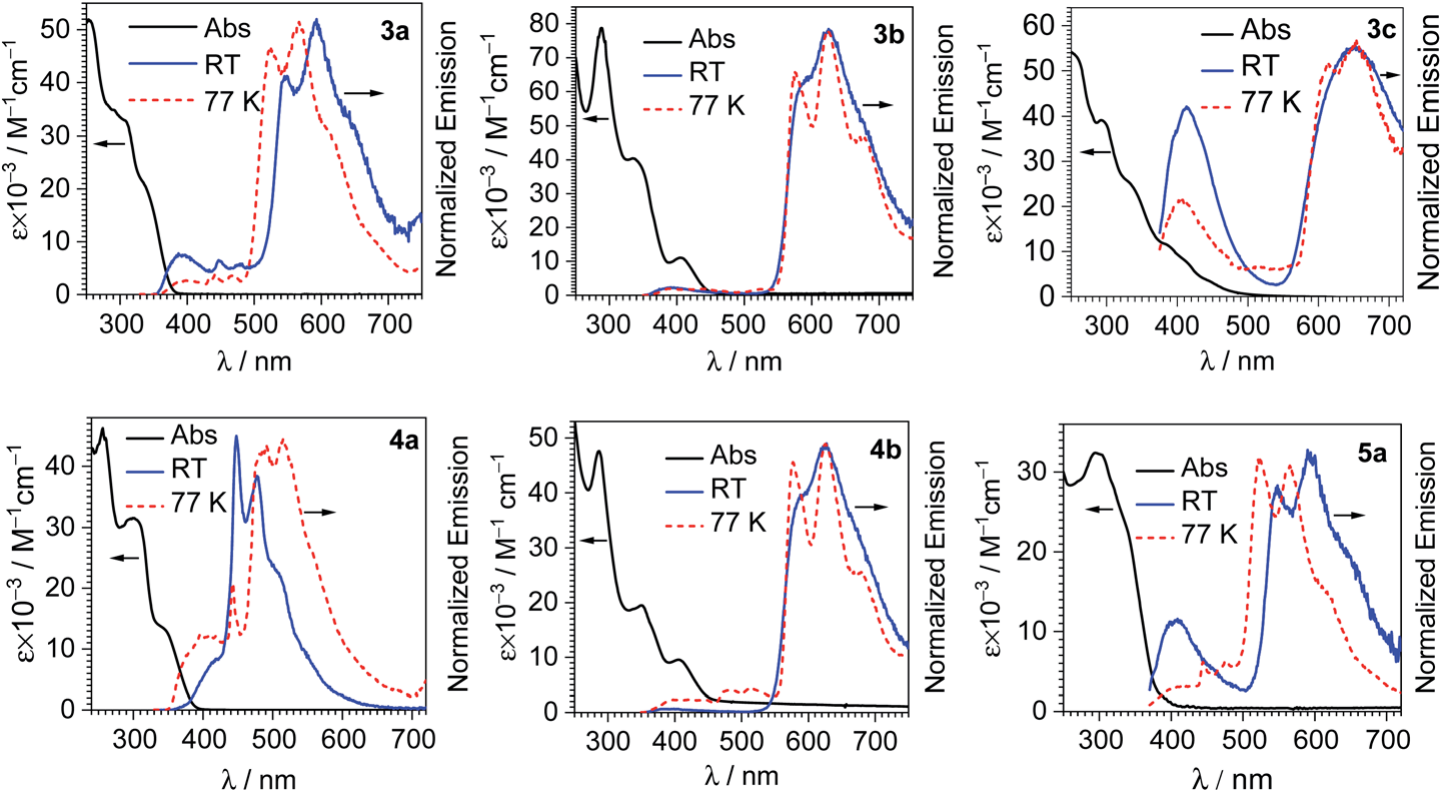
Overlaid UV-vis absorption and photoluminescence spectra of the iridium-coumarin complexes. UV-vis absorption (solid black line) and room-temperature photoluminescence (blue solid line) were both recorded in CH_2_Cl_2_ at 293 K, and photoluminescence at 77 K (red dashed line) was measured in 1 : 3 (v/v) CH_2_Cl_2_/toluene.

Fig. 2 shows overlaid UV-vis absorption and photoluminescence spectra of each iridium-coumarin compound, with the numerical data for the photoluminescence summarized in Table 1. PL spectra were recorded both at room temperature in CH_2_Cl_2_ and at 77 K in 1 : 3 CH_2_Cl_2_/toluene glass; spectra at 77 K are shown in Fig. 2 as well, with the data summarized in Table S2 of the ESI.† The iridium-coumarin complexes have near-UV absorption in the same regions as free coumarins C-1 and C-2, overlapped with other strong absorption bands that are likely π→π* transitions from the C^N ligands chelated to iridium. Metal-to-ligand charge transfer (MLCT) bands originating from an Ir(5d)→C^N(π*) transitions are clearly observed in the complexes with the more conjugated C^N ligands piq and pphen. These broad bands occur at *ca.* 410 nm in piq complexes 3b and 4b and *ca.* 420 nm in pphen complex 3c.

**Table 1:** Summary of photoluminescence properties of the iridium-coumarin complexes. Room-temperature photoluminescence spectra were measured in CH_2_Cl_2_ at 293 K. Samples for steady-state photoluminescence measurement were excited at 310 nm, and for lifetime measurements at 330 nm. For each set of PL data, the wavelength with the maximum intensity is labelled with “(max).” Both fluorescent (*τ*_F_) and phosphorescent (*τ*_P_) lifetimes are reported, where applicable

	*λ*_em_/nm (293 K)	*Φ*_PL_	*τ*_F_/*n*_s_	*τ*_P_/*μ*_s_
3a	395, 447, 477, 547, 592 (max), 648	N.D.	9.0	N.D.
3b	587, 627 (max)	0.17	N.A.	6.4
3c	413, 650 (max)	0.022^a^, 0.086^b^	2.1	2.7
4a	415, 448 (max), 478, 506	0.027	N.D.	1.6
4b	591, 626 (max)	0.22	N.A.	6.5
5a	407, 550, 592 (max), 650	0.0061^a^, 0.016^b^	10.8	N.D.

a Fluorescence quantum yield.

b Phosphorescence quantum yield.

Among the six compounds, there are three distinct patterns for the room-temperature photoluminescence:

(1) In piq complexes 3b and 4b, the only appreciable photoluminescence is phosphorescence from the bis-cyclometalated iridium fragment. The emission both at 298 and 77 K is almost identical to the previously described model complex [Ir(piq)_2_(CNAr^dmp^)(pyridine)](PF_6_).^35^ The lifetimes of 3b and 4b, *c.a.* 6 μs, are likewise similar to the model complex, and photoluminescence quantum yields (*Φ*_PL_) are about a factor of two higher, 0.17 (3b) and 0.22 (4b), *vs.* 0.096 in the model complex. Because these two compounds only exhibit phosphorescence and no fluorescence, they are not suitable candidates for ratiometric oxygen sensing.

(2) In complexes 3c and 4a, there is dual emission involving fluorescence from the coumarin and phosphorescence from the [Ir(C^N)_2_]^+^ moiety. The fluorescence occurs at similar wavelengths as the free coumarins (see Fig. S25 and S26†), and the phosphorescence is dictated by the cyclometalating ligand. In 4a the coumarin fluorescence overlaps strongly with the phosphorescence, appearing as a shoulder on the high-energy side of the phosphorescence vibronic progression. This substantial overlap between fluorescence and phosphorescence means that 4a would not function well as a ratiometric sensor. The phosphorescence spectrum and lifetime (1.6 μs) in 4a strongly resemble those of an [Ir(F_2_ppy)_2_(CNAr^dmp^)(pyridine)](PF_6_) model complex,^35^ further confirming this assignment. In pphen complex 3c, the blue fluorescence from the coumarin (*λ*_max_ = 413 nm) is well-separated from the deep red phosphorescence (*λ*_max_ = 650 nm), with no appreciable overlap between the two signals. The good resolution between the two signals allowed us to separately integrate the fluorescence and phosphorescence bands and determine quantum yields for each. With excitation at 310 nm, the fluorescence quantum yield is 0.022, and the phosphorescence quantum yield is 0.086. The large separation between the two bands also makes complex 3c ideally suited for ratiometric sensing, as described below.

The UV-vis absorption and PL spectra of 3c also indicate the possibility of Förster Resonance Energy Transfer (FRET) in this complex, with the coumarin fluorescence overlapping the ^1^MLCT absorption from iridium. As further experimental evidence for this phenomenon, the coumarin fluorescence quantum yield and fluorescence lifetime decrease when the coumarin is coordinated to iridium in 3c. The fluorescence quantum yields of C-1 and 3c are 0.083 and 0.022, respectively, and the fluorescence lifetime (3.4 ns in C-1*versus* 2.1 ns in 3c) is attenuated as well in 3c. That said, since ^1^MLCT population is followed by rapid intersystem crossing to ^3^MLCT we can't spectroscopically monitor FRET directly, and there are other pathways that could populate ^3^MLCT, but the spectral data suggest FRET may be occurring.

(3) In the other two F_2_ppy complexes 3a and 5a, dual luminescence with some blue fluorescence from the coumarin is likewise observed. However, the phosphorescence in these compounds, with two vibronic maxima at *ca.* 550 and 590 nm, occurs at much longer wavelength than typically observed in [Ir(F_2_ppy)_2_]^+^ complexes. This structured luminescence in 3a and 5a is ascribed to phosphorescence originating from a coumarin-centered triplet state. Phosphorescence occurring from coumarin has been observed, for example in platinum acetylide,^41^ cyclometalated Pt(II)^42^ and cyclometalated Ir(iii) complexes,^43–45^ and normally arises from triplet intraligand excited states (^3^IL) that give red emission. The phosphorescence in 3a and 5a occurs in a similar region of the spectrum as these literature precedents. Moreover, the expected blue phosphorescence from the ^3^MLCT state of the [Ir(F_2_ppy)_2_]^+^ fragment is absent, likely quenched by energy transfer from the higher energy ^3^MLCT state to the lower energy coumarin ^3^IL state. Based on these observations, we conclude that the phosphorescence in 3a and 5a is assigned to a coumarin-centered ^3^IL state.

Overlaid UV-vis absorption and excitation spectra of each complex are shown in Fig. S27–S32 of the ESI,† and they reveal additional insights into the excited-state dynamics. Stated briefly, to observe coumarin fluorescence (all but 3b and 4b), the coumarin moiety must be directly excited. Excitation anywhere in the UV-vis absorption window gives rise to phosphorescence, and when monitoring the excitation spectrum at the phosphorescence maximum the UV-vis absorption and excitation spectra match well. In other words, for the T_1_ state Kasha's rule is followed. One interesting observation noted above is that in F_2_ppy complexes 3a and 5a, which both use the 3-pyridyl-substituted coumarin, phosphorescence occurs from the coumarin triplet state, whereas in 4a, where the longer carboxy-pyridine linker is used, phosphorescence occurs from the iridium center. This phenomenon underscores the importance of the linker between the fluorophore and phosphor in determining the energy-transfer dynamics and suggests that with the longer linker in 4a triplet energy transfer to the coumarin is not as rapid, and all phosphorescence occurs from the iridium center.

### Ratiometric oxygen sensing

**Fig. 3:**
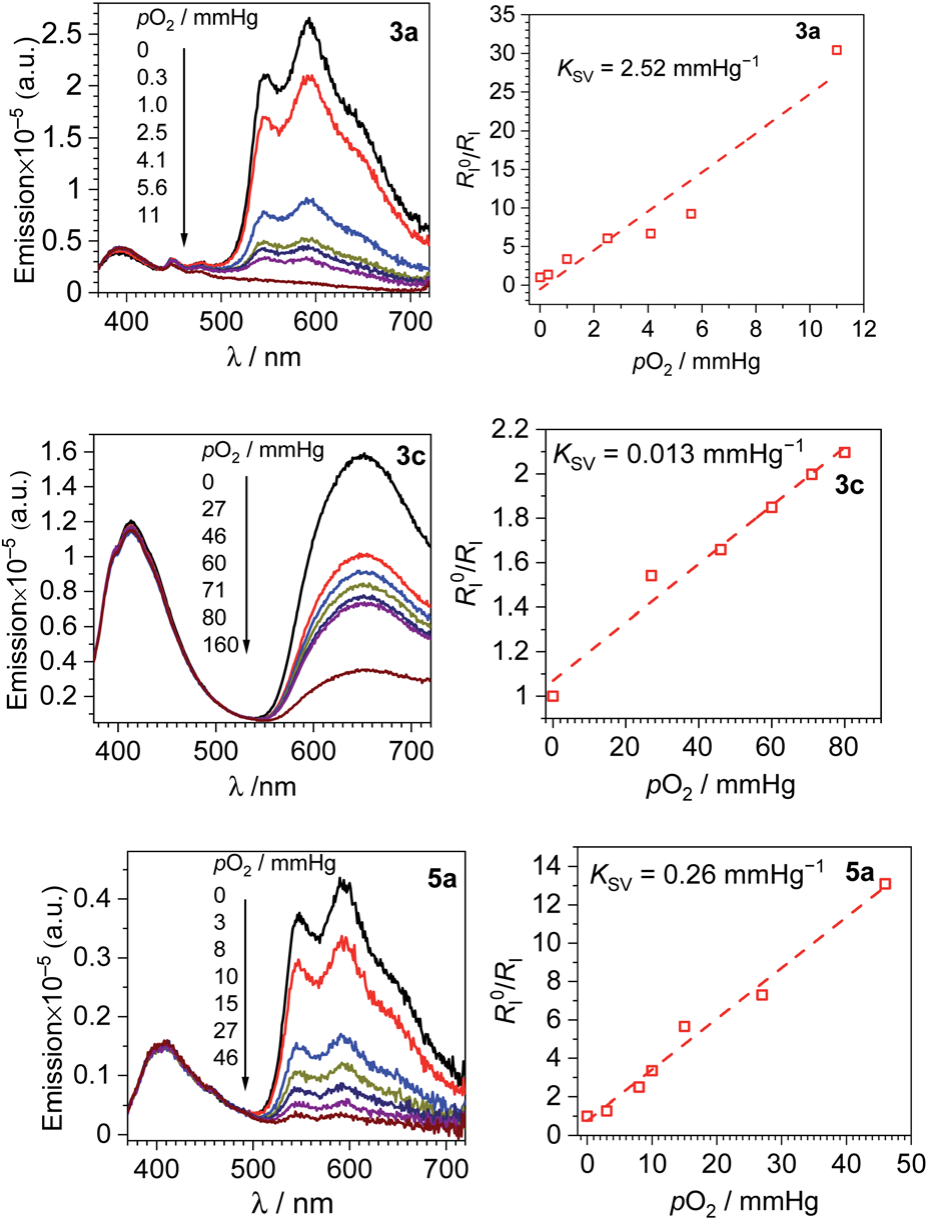
Spectral response to increasing oxygen concentration (left column), Stern–Volmer oxygen quenching studies (right column) for 3a, 3c and 5a. All data were recorded in CH_2_Cl_2_ at 293 K.

F_2_ppy complexes 3a and 5a and pphen complex 3c exhibit clear dual emission and hence were chosen for ratiometric oxygen sensing. A qualitative assessment of all six complexes' response to oxygen was performed by preparing each sample in a nitrogen-filled glovebox, the photoluminescence spectra observed, then the spectra taken again after equilibration in air. The spectra of each complex in N_2_-saturated *versus* aerated CH_2_Cl_2_ solutions can be found in Fig. S33–S38 of the ESI.† For the dual-emitting compounds there is no change in coumarin fluorescence upon exposure to air, while in all compounds, phosphorescence is quenched. A quantitative assessment was performed on dual-emitting compounds 3a, 5a and 3c by taking photoluminescence spectra at increasing *p*O_2_ levels, either until phosphorescence was completely quenched or until atmospheric levels of oxygen (*ca.* 160 mm Hg) were present. Fig. 3 (left column) shows the spectral change for each complex with increasing *p*O_2_ levels, which clearly shows quenching of the longer-wavelength phosphorescence with no change in the coumarin's fluorescence intensity. All three complexes are capable of sensing partial pressures of oxygen below atmospheric content (pO_2_ ≤ 160 mm Hg), meaning they can be used in hypoxic environments.

A numerical description of the oxygen sensing comes from Stern–Volmer analysis. Eqn (1) shows the Stern–Volmer relationship, where *K*_SV_ is the Stern–Volmer constant, *p*O_2_ is the oxygen partial pressure, *k*_q_ is the quenching rate constant, and *τ*_0_ and *τ* are the lifetimes without and with oxygen present, respectively.^46,47^ In complexes 3a and 5a the photoluminescence is too weak and the lifetimes too long to determine accurate values on our instrumentation, so we analyzed the data with a previously reported modified Stern–Volmer equation that is appropriate for ratiometric sensors.^5^ In this method *R*_I_ describes the intensity ratio of phosphorescence to fluorescence, with *R*_I_^0^ describing this ratio in the absence of O_2_. Eqn (2) shows this modified Stern–Volmer relationship, using *R*_I_^0^/*R*_I_ as the dependent variable with *K*_SV_ and *p*O_2_ defined the same as in eqn (1).^5^1
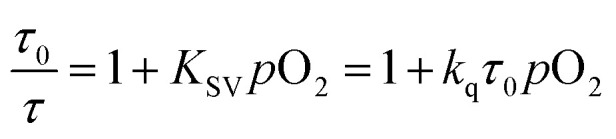
2
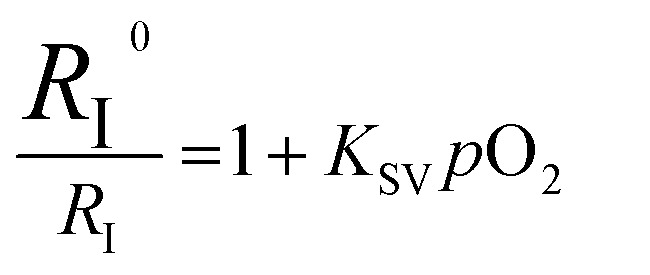


The Stern–Volmer constant *K*_SV_ can be obtained from the slope of the linear fit, and all three compounds in Fig. 3 give very different *K*_SV_ values, indicating different sensitivities to O_2_. *K*_SV_ for pphen complex 3c is 1.3 × 10^−2^ mmHg^−1^, slightly smaller than those of our first-generation sensors (*K*_SV_ = 3.0–8.1 × 10^−2^ mmHg^−1^),^34^ but same order of magnitude. For complexes 3a and 5a where the phosphorescence arises from the coumarin triplet state, the oxygen sensitivity is much higher, and *K*_SV_ is one or two orders of magnitude higher than that of 3c, measured at 0.26 (5a) and 2.5 (3a) mmHg^−1^. For complex 3c the inherent phosphorescence lifetime *τ*_0_ is known (2.7 μs, see Table 1), allowing us to use eqn (1) to determine a bimolecular quenching rate constant, *k*_q_, of 4.8 × 10^3^ s^−1^mm Hg^−1^, equivalent to 3.3 × 10^8^ s^−1^M^−1^ using the known solubility of oxygen gas in dichloromethane.^48^ This *k*_q_ value approaches the diffusion limit which suggests that 3c is efficiently quenched by O_2_, and is within the range of *k*_q_ values observed in our first-generation Ir-BODIPY sensors.^34^ Without available lifetime values we cannot determine *k*_q_ values for 3a and 5a, but in most cases oxygen quenching of triplet states is near the diffusion limit,^49^ so if we assume that *k*_q_ spans the range of 10^8^–10^9^ s^−1^M^−1^, we can estimate that the excited-state lifetime for 3a is in the range of 10^−4^ to 10^−3^ s, and that of 5a about an order of magnitude shorter in the range of 10^−5^ to 10^−4^ s. Both of these are substantially longer than that of 3c, consistent with the organic ^3^(π→π*) nature of the emissive state in 3a and 5a, *versus* the significant Ir(5d)→C^N(π*) ^3^MLCT character in 3c.

To better quantify and compare the dynamic ranges of the sensors, here we define a parameter abbreviated as *p*(O_2_)_90%_, the value of *p*(O_2_) needed to reach 90% quenching. In these sensors the good signal resolution allows *R*_I_ to approach 0, so *p*(O_2_)_90%_ is defined as the pressure where *R*_I_^0^/*R*_I_ = 10. Using the best-fit Stern–Volmer lines in Fig. 3, we extrapolate *p*(O_2_)_90%_ values of *ca.* 4 mm Hg (3a), 36 mm Hg (5a), and 690 mm Hg (3c). Thus, all three sensors operate under very different dynamic ranges, giving good sensitivity to small (3a), intermediate (5a), and large ranges of *p*O_2_ (3c). The slightly smaller *K*_SV_ value and much improved signal resolution in 3c combine to give a much larger dynamic range when compared to our previous Ir-BODIPY sensors, by at least a factor of 10 in terms of the estimated *p*(O_2_)_90%_. The superior signal resolution in 3c is critical, as the smaller *K*_SV_ value accounts for only about a factor of 2.5 difference in dynamic range. All these metrics show that, using our synthetic approach that allows us to easily join different fluorophores and phosphors, we can dial in a wide range of sensor attributes, allowing us to optimize the characteristics for a specific application.

Whereas the phosphorescence of 5a is quenched in water, the other two ratiometric sensors (3a and 3c) can operate in aqueous solution. Qualitative oxygen quenching experiments for these two sensors were performed in deionized water, recording spectra in nitrogen-saturated and aerated solutions, as shown as Fig. S39 and S40 of the ESI.† The solubility of oxygen in water^51^ is about 30× lower than in dichloromethane,^48^ so the ratiometric response will be attenuated in water at parity of *p*O_2_. The high sensitivity of 3a to O_2_ leads to significant quenching of the phosphorescence band in the presence of air (Fig. S39†). However, 3c has a much shorter phosphorescence lifetime, and when coupled with the much lower solubility of O_2_ in water, leads to minimal quenching of the aerated solution (Fig. S40†). This outcome further underscores the importance of designing sensors with a range of photophysical characteristics, since the same sensor may not operate the same in two different environments given the disparate oxygen solubilities.

**Fig. 4:**
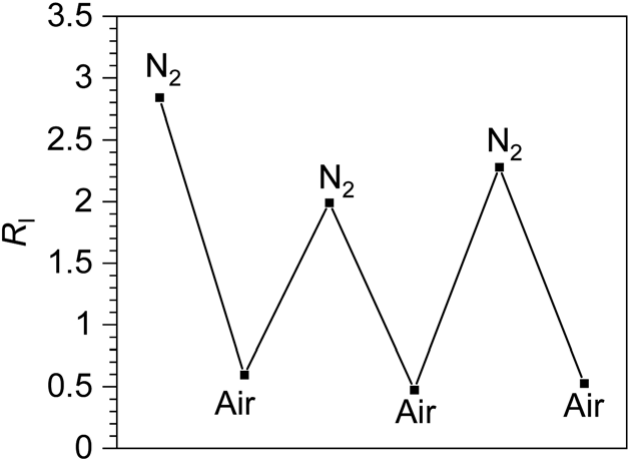
Reversible ratiometric sensing of O_2_ by complex 3c. The first N_2_-saturated sample was prepared in the glovebox and cycled between air and nitrogen atmospheres by exposing to air followed by freeze–pump–thaw degassing (3×). The *y*-axis shows *R*_I_, the ratio of phosphorescence intensity to fluorescence intensity, for each cycle (see Fig. S41†).

To further evaluate the capabilities of 3c, the reversibility of oxygen sensing was tested. A solution of 3c was prepared in a nitrogen atmosphere and the photoluminescence spectrum recorded. The same sample was then exposed to air and re-measured. After this first cycle, the sample was deoxygenated by freeze-pump thaw, and two additional N_2_-air cycles were recorded. The photoluminescence data is plotted in Fig. S41 in the ESI,† and Fig. 4 below plots the ratiometric response of this cycling test. In Fig. 4, the ratio of phosphorescence intensity to fluorescence intensity, *R*_I_, is plotted for each cycle. Although *R*_I_ does not completely return to its original value after the first cycle, likely indicating that our freeze–pump–thaw protocol is not perfectly effective at removing air, the sensor is clearly reversible. The *R*_I_ value cycles between high (N_2_) and low (air) values throughout the experiment and suggests that 3c can be used for temporal monitoring of O_2_.

The observed reversibility in Fig. 4 suggests that 3c, and by extension the other ratiometric sensors, operate by the conventional O_2_ sensing mechanism where the T_1_ state is quenched by triplet oxygen (^3^O_2_) *via* triplet–triplet annihilation, reducing the phosphorescence intensity and generating singlet oxygen (^1^O_2_) in the process. To test this supposition further, singlet oxygen generated by 3a, 3c, and 5a was quantified by measuring the characteristic emission of ^1^O_2_ at ∼1270 nm in comparison to a standard of [Ru(bpy)_3_]^2+^ (*Φ*_Δ_ = 0.57 in DMF).^50^ The ^1^O_2_ emission was observed in all cases (Fig. S42–S45†), and *Φ*_Δ_ values of 0.13, 0.26, and 0.22 were measured for complexes 3a, 3c, and 5a, respectively. While ^1^O_2_ generation is not particularly efficient in these compounds, and we can't rule out other reactive oxygen species (ROS) being formed alongside ^1^O_2_, these experiments do show that triplet–triplet annihilation is a dominant sensing pathway in these compounds.

Finally, to test for photostability, air-saturated samples of the three sensors 3a, 3c, and 5a were subjected to 310 nm irradiation for prolonged periods of time, and 3c was also exposed to a blue LED light since this compound absorbs in the visible region. Photoluminescence spectra were recorded at each time point, with the data summarized in Fig. S46–S49 of the ESI.† In all cases we see gradual increase of the coumarin fluorescence intensity during irradiation, and in the case of 3c a slight decrease in phosphorescence intensity is observed over the 2 hour time period. A likely explanation of these results is gradual photodissociation of the pyridyl-coumarin ligand, which results in an increase in fluorescence intensity from the free coumarin.

## Conclusions

To summarize, we have synthesized a set of cyclometalated iridium-coumarin dyads applied as ratiometric oxygen sensors. The complexes are prepared using relatively simple synthetic methods, allowing ready access to several structural variants. The compounds have diverse photoluminescence profiles, determined by the choice of cyclometalating ligand on iridium and the linker between the coumarin and the iridium center. In some cases, only phosphorescence from the cyclometalated iridium center is observed, and in others dual luminescence involving blue fluorescence from the coumarin and longer-wavelength phosphorescence occurs, the latter arising either from an iridium-centered ^3^MLCT state or a coumarin-centered ^3^(π→π*) state. Three of the six complexes exhibit well-resolved dual emission, and their oxygen-sensing attributes are distinctly improved in relation to our first-generation sensors. In all cases clear resolution between the fluorescence and phosphorescence signals is observed, with no overlap. The sensitivities and dynamic ranges of the sensors span two orders of magnitude, showing that the structural modifications enabled by our synthetic approach can have dramatic consequences on the sensing profile. In complex 3c, where phosphorescence arises from a ^3^MLCT state centered on the [Ir(pphen)_2_]^+^ phosphor, the comparatively small Stern–Volmer constant (*K*_SV_) and large separation between fluorescence and phosphorescence enables reversible oxygen detection over a wide range of partial pressures, substantially improved over our first-generation sensors where the green fluorescence and red phosphorescence overlapped. In compounds 3a and 5a, the coumarin-centered phosphorescence is apparently much longer lived, leading to much higher sensitivity to low O_2_ levels. Thus, these latter sensors are ideally suited to applications where accurate detection of small O_2_ partial pressures is needed. This work shows the versatility of our approach in designing effective ratiometric oxygen sensors. These sensors have limited potential for applications in cellular environments due to their modest quantum yields and the short wavelengths in the UV region. To improve photoluminescence quantum yields, which are all less than 0.1 in this class of sensors, we will leverage our group's previous insights into the design of red and near-infrared phosphors with high quantum yields.^52,53^ Combining these design elements for efficient red and near-infrared phosphorescence with visible-absorbing fluorophores that we have previously used^34,36^ will allow us to design brighter sensors that can be excited in the visible region.

## Author contributions

Yanyu Wu: conceptualization, formal analysis, investigation, visualization, writing – original draft, writing – review & editing. Gregory D. Sutton: formal analysis, investigation, visualization, writing – original draft, writing – review and editing. Michael D. S. Halamicek: formal analysis, investigation, writing – review & editing. Xinxin Xing: formal analysis, investigation, writing – review & editing. Jiming Bao: formal analysis, supervision, writing – review & editing. Thomas S. Teets: conceptualization, formal analysis, project administration, supervision, visualization, writing – review & editing.

## Conflicts of interest

There are no conflicts to declare.

## Supplementary Material

SC-013-D2SC02909J-s001

SC-013-D2SC02909J-s002
