# Supplementary material for: Cyclometalated iridium-coumarin ratiometric oxygen sensors: improved signal resolution and tunable dynamic ranges
Source: Chem Sci. 2022 Jul 15;13(30):8804–12. doi: 10.1039/d2sc02909j (PMC9350586; doi:10.1039/d2sc02909j)
Supplement: SC-013-D2SC02909J-s001 [file SC-013-D2SC02909J-s001.pdf]

Electronic Supplementary Material (ESI) for Chemical Science.
This journal is © The Royal Society of Chemistry 2022

Electronic Supplementary Information for

Cyclometalated Iridium-Coumarin Ratiometric Oxygen Sensors: Improved Signal Resolution and Tunable Dynamic Ranges

Yanyu Wu, Gregory D. Sutton, Michael Halamicek, Xinxin Xing, Jiming Bao and Thomas S. Teets*

Department of Chemistry, University of Houston,
3585 Cullen Blvd. Room 112, Houston, TX 77204-5003, USA
email: tteets@uh.edu

Index	*Page*
Experimental Section	S2–S6
X-ray crystallography data for complexes **3a – 5a**	S7
NMR spectra of all new complexes	S8–S25
High resolution mass spectrometric analysis of all new complexes	S26–S31
UV-vis absorption and photoluminescence spectra of free coumarins	S32
Summary of photoluminescence data recorded at 77 K	S33
Overlaid UV-vis absorption and excitation spectra of iridium-coumarin complexes	S34–S36
Photoluminescence spectra of iridium-coumarin complexes in deaerated and aerated solutions	S37–S40
Reversibility tests for complex **3c**	S41
Singlet oxygen emission spectra for $[Ru(bpy)_3]^{2+}$ and complexes **3a**, **3c**, and **5a**	S42–S43
Photostability tests for complexes **3a**, **3c**, and **5a**	S44–S45

Experimental Section

Materials. All chemicals were purchased from commercially available sources and were used without further purification unless otherwise specified. Solvents were deoxygenated and dried using a Grubbs Solvent Purification System. Iridium precursor complexes **1a**, **1b**, **2a**, and **2b** were synthesized following previously reported methods.[1,2] Complexes **1c** and **2c** were prepared using analogous approaches.

Physical methods. ^{1}H, ^{19}F, and $^{13}C\{^{1}H\}$ NMR spectra were recorded at room temperature on a JEOL ECA-400, ECA-500 or ECA-600 NMR spectrometer. UV–vis absorption spectra were recorded in screw-capped 1 cm quartz cuvettes using an Agilent Carey 8454 UV–vis spectrophotometer. Emission and excitation spectra were obtained using a Horiba FluoroMax-4 spectrofluorometer. Room-temperature emission samples were housed in 1 cm quartz cuvettes with septum-sealed screw caps and the low-temperature emission spectra were recorded in a custom quartz EPR tube with high-vacuum valve immersed in liquid nitrogen using a finger Dewar. Samples for emission and excitation measurements were prepared inside a nitrogen-filled glovebox using dry and deoxygenated solvents to exclude air. Luminescence lifetimes were measured with a Horiba DeltaFlex Lifetime System, using pulsed diode excitation and excited at 330 nm. Emission wavelengths were selected by using appropriate long-pass filters, and the decay trace was fitted using the instrument's analysis software or the software Origin 2020b. Emission quantum yields for complex **4a** were measured with respect to a standard of quinine sulfate in 0.05 M sulfuric acid having a reported quantum yield (Φ_F) of 0.52[3], while quantum yields for complexes **5a**, **3b**, **4b** and **3c** were measured relative to tetraphenylporphyrin, which has a reported Φ_F of 0.11[4]. The quantum yields of the Ir-coumarin conjugates (Φ_x) was calculated using Equation 5 below, where Φ_{st} = the quantum yield of the standard, m_x = the slope of emission intensity versus absorbance for the samples, m_{st} = the slope of emission intensity versus absorbance for the standard compound, and η_x and η_{st} are the refractive indexes of the solvents of the sample and standard, respectively.

$$\Phi_x = \Phi_{st}\left[\frac{m_x}{m_{st}}\right]\left[\frac{\eta_x}{\eta_{st}}\right]^2 \quad (5)$$

Oxygen quenching experiments. Iridium-coumarin complex **3a**, **3c**, or **5a** was dissolved in dichloromethane in a nitrogen-filled glovebox. For complexes **3a** and **5a,** the stock solutions were further diluted in quartz cuvettes to concentrations of 1.0×10^{-5} M. The emission spectra for **3a** and **5a** were recorded under nitrogen atmosphere (pO_2 = 0 mmHg), then aliquots of air were added to each cuvette (1–5 µL for **3a** and 10–50 µL for **5a**), and the emission spectra were recorded after each addition using an excitation wavelength of 310 nm until phosphorescence was completely quenched. The change in the ratio of phosphorescence to fluorescence intensity was used alongside the change in oxygen partial pressure to get Stern-Volmer quenching constants (K_{SV}) for these two complexes. In the case of **3c**, the stock solution was diluted in a quartz cuvette to a concentration of 3.0×10^{-6} M. The emission spectrum was recorded under nitrogen atmosphere (pO_2 = 0 mmHg), then 50–100 µL aliquots of air were added to the cuvette, and the emission spectra were recorded after each addition using an excitation wavelength of 310 nm until atmospheric levels of oxygen were reached (pO_2 = 160 mmHg). Since **3c** has adequately observable photoluminescence and a short enough lifetime to get accurate measurements on our instrument, the lifetime was also taken under nitrogen atmosphere and after each addition of air using 330 nm excitation for the lifetime decay. The Stern-Volmer quenching constant K_{SV} and the quenching rate constant (k_q) were calculated for complex **3c**.

Reversibility experiments. A sample of iridium-coumarin complex **3c**, was dissolved in deoxygenated dichloromethane in a nitrogen-filled glovebox. The stock solution was further diluted in a quartz cuvette to a concentration of ca. 10^{-6} M. N_2-saturated emission was recroded between 350–700 nm using an excitation wavelength of 310 nm, and the emission was repeated after fully aerating the sample. To test for reversibility, the solution was degassed via freeze-pump-thaw, added back to the cuvette inside the glovebox, and the emission measurement was repeated.

Detection of singlet oxygen. Iridium-coumarin complexes **3a**, **3c**, and **5a** were dissolved in dichloromethane under aerated conditions. Stock solutions of each complex were further diluted in quartz cuvettes to concentrations on the order of 10^{-5} M, corresponding to absorbance values of 0.3 at the excitation wavelength of 365 nm. A standard of $[Ru(bpy)_3](PF_6)_2$ was prepared in DMF and likewise diluted to give an absorbance value of 0.3 at 365 nm. A 365-nm Thorlabs 500 mW LED was used for excitation and a Horiba Jobin Yvon

iHR 320 spectrometer equipped with a Symphony liquid nitrogen cooled InGaAs NIR linear array detector was used to obtain singlet oxygen emission spectra. To get sufficient signal-to-noise, an integration time of 240 seconds (4 minutes) was used and 2 scans were completed for each sample for a total of 480 seconds (8 minutes). A 1000-nm long pass filter was used, and the same path length of 1 cm was also used for every sample. To calculate the singlet oxygen quantum yield (Φ_Δ), the samples were compared to the $[Ru(bpy)_3]^{2+}$ standard which has a known Φ_Δ of 0.57 in DMF.[5] A modified quantum yield expression[6] (Equation S1 below) was used, where $\Phi_{\Delta S}$ is the quantum yield of the sample, $\Phi_{\Delta r}$ is the quantum yield of the reference, I_r and I_s are the fractions of incident light absorbed by the reference and sample, respectively, given by $1-10^{-A}$ at the excitation wavelength of 365 nm, $I_{\Delta r}$ and $I_{\Delta S}$ are the integrated singlet oxygen emission intensities of the reference and sample, respectively, and τ_r and τ_s are the singlet oxygen phosphorescence lifetimes in the reference and sample solvents, respectively. The value of τ_r in DMF is known to be 12.1 μs[7], while the value of τ_s in CH_2Cl_2 is known to be 91 μs.[8]

$$\Phi_{\Delta s} = \Phi_{\Delta r} \frac{I_r}{I_s} \frac{I_{\Delta s}}{I_{\Delta r}} \frac{\tau_r}{\tau_s} \qquad \text{(S1)}$$

Photostability experiments. Iridium-coumarin complexes **3a**, **3c**, and **5a** were dissolved in dichloromethane under aerated conditions. Stock solutions of each complex were further diluted in quartz cuvettes to concentrations of 2.0×10^{-6} M. To test the photostability of each complex under UV light, the complexes were irradiated in the fluorimeter directly using 310 nm irradiation in 15-minute increments for a total of 2 hours. In each UV irradiation experiment the monochromator slit widths were opened to 10 nm during irradiation and were set back to 5 nm to collect emission spectra. Since **3c** also has absorption in the visible range, photostability tests were likewise performed by irradiation using a glass bowl wrapped with blue LED strips, purchased from Creative Lighting Solutions (Model: Sapphire Blue LED Tape −12vdc), and wrapped on the outside with aluminum foil. To maintain irradiation temperature, the vessel was filled with water. During the experiments involving irradiation with blue LEDs the water bath reached a maximum temperature of 30 ºC. For all experiments, emission spectra were collected by exciting at 310 nm and recording the emission spectrum from 350–700 nm. The same path length of 1 cm was used in each case.

X-ray crystallography details. Single crystals were grown by vapor diffusion or liquid-liquid layering. Crystals were mounted on a Bruker Apex II three-circle diffractometer using MoKα radiation (λ = 0.71073 Å). The data was collected at 123(2) K and was processed and refined within the APEXII software. Structures were solved by intrinsic phasing in SHELXT and refined by standard difference Fourier techniques in the program SHELXL.[9] Hydrogen atoms were placed in calculated positions using the standard riding model and refined isotropically; all non-hydrogen atoms were refined anisotropically. The crystal of **3a** was a non-merohedral twin, so for this crystal two unit cell domains were identified in the program CELL_NOW and the data was integrated against both components. The program TWINABS was used to perform the absorption correction, and HKLF5 refinement was performed to refine the structure against both domains. The structure of **4a** included a disordered benzene solvent molecule, which was modeled as a two-part disorder. The bond distances and angles in all disordered parts were restrained using SADI commands, and the ellipsoid parameters were restrained with the rigid-bond restraints SIMU and DELU. The structure of **5a** included heavily disordered solvent electron density that could not be satisfactorily refined, necessitating the use of the SQUEEZE function in PLATON.[10] Crystallographic details are summarized in Table S1.

Syntheses

Synthesis of coumarin C-1. This compound was prepared as previously described.[11] A mixture of 3-pyridylacetic acid (1.10 g, 6.4 mmol), salicylaldehyde (0.5 mL, 3.5 mmol), acetic anhydride (1.2 mL, 11 mmol) and triethylamine (1 mL) was heated at 185 ºC for 3 hours. Afterwards, the reaction was cooled down and washed with DI-water and diethyl ether obtaining the final product as white powder. The spectral data matches that previously reported for this compound. (Yield: 505 mg, 65%) ^{1}H NMR (600 MHz, $CDCl_3$): δ = 8.88 (s, 1H), 8.65 (s, 1H), 8.15 (d, 1H, J = 7.8 Hz), 7.90 (s, 1H), 7.58 (t, 2H, J = 9.0 Hz), 7.39–7.41 (m, 2H), 7.34 (t, 1H, J = 7.5 Hz).

Synthesis of coumarin C-2. This product was prepared as previously described.[12] Inside the glovebox, a solution containing coumarin-3-carboxylic acid (100 mg, 0.53 mmol), 4-hydroxypyridine (53 mg, 0.56 mmol) and dimethylaminopyridine (6 mg) in anhydrous DCM (100 mL) was combined with EDC·HCl (110 mg,

0.57 mmol) and allowed to stir at room temperature overnight. Afterwards, the mixture was washed with DI water and the organic layer was collected and dried over $MgSO_4$, which was then filtered, and the filtrate was dried over vacuum. The obtained white solid was washed with ether and ethanol and recrystallized from chloroform and diethyl ether. (Yield: 73 mg, 51%) ^{1}H NMR (400 MHz, $CDCl_3$): δ = 8.88 (s, 1H), 7.87 (d, 2H, J = 7.2 Hz), 7.72–7.76 (m, 2H), 7.42–7.47 (m, 2H), 6.68 (d, 2H, J = 7.6 Hz).

Synthesis of 3a. Inside the glovebox, complex **2a** (100 mg, 0.14 mmol) was dissolved in 10 mL of CH_2Cl_2 and combined with $AgPF_6$ (34 mg, 0.13 mmol) and 3-(3-pyridyl)coumarin (**C-1**) (31 mg, 0.14 mmol), which turned into a cloudy yellow suspension. The mixture was stirred overnight at room temperature. Then the reaction mixture was filtered, and the solvent was removed under vacuum to obtain an oily yellow material. The final product was obtained after silica gel column chromatography eluting with CH_2Cl_2/Ethyl acetate (7:3) and recrystallization from CH_2Cl_2/Et_2O as light-yellow powder. (Yield: 52 mg, 36%) ^{1}H NMR (600 MHz, CD_3CN): δ = 9.40 (d, 1H, J = 5.4 Hz), 9.11 (s, 1H), 8.69 (s, 1H), 8.43 (t, 2H, J = 8.7 Hz), 8.34 (d, 1H, J = 9.0 Hz), 8.26 (d, 1H, J = 7.8 Hz), 8.13 (t, 1H, J = 8.1 Hz), 8.08 (t, 1H, J = 7.8 Hz), 7.97 (s, 1H), 7.66 (t, 2H, J = 7.5 Hz), 7.55 (t, 1H, J = 6.3 Hz), 7.45 (t, 1H, J = 6.6 Hz), 7.40 (t, 3H, J = 7.8 Hz), 7.25 (t, 1H, J = 7.8 Hz), 7.13 (d, 2H, J = 7.2 Hz), 6.64–6.71 (m, 2H), 5.85 (d, 1H, J = 7.8 Hz), 5.74 (d, 1H, J = 6.6 Hz), 2.09 (s, 6H). ^{19}F NMR (564 MHz, CD_3CN): δ = –72.35 (d, 6F, J = 705.0 Hz, PF_6), –106.35 (q, 1F, J = 10.2 Hz, F_2ppy), –107.65 (q, 1F, J = 10.2 Hz, F_2ppy), –108.31 (t, 1F, J = 13.3 Hz, F_2ppy), –109.65 (t, 1F, J = 13.0 Hz, F_2ppy). $^{13}C\{^1H\}$ NMR (151 MHz, $CDCl_3$): δ = δ = 165.8, 164.4 (dd, J_{CF} = 260 Hz, 12 Hz), 164.1 (d, J_{CF} = 7.6 Hz), 162.6 (dd, J_{CF} = 230 Hz, 12.8 Hz), 161.6 (dd, J_{CF} = 263 Hz, 12 Hz), 161.2 (dd, J_{CF} = 334 Hz, 12.8 Hz), 160.6, 153.9, 150.6, 144.3, 144.2, 144.1, 144.01, 143.99, 142.7, 139.9, 139.7, 139.0, 135.6, 134.4, 132.8, 130.0, 129.6, 128.3, 127.6, 127.5, 126.1, 125.4, 125.3, 124.52, 124.48, 124.3, 124.2, 124.0, 122.4, 119.4, 116.4, 114.3, 114.1, 113.0, 112.9, 101.0 (t, J_{CF} = 30.0 Hz), 99.6 (t, J_{CF} = 30.0 Hz), 18.3. HRMS: *m/z* calculated for $C_{45}H_{30}F_{10}IrN_4O_2P$ $[M-PF_6]^+$: 927.1929, found: 927.1943.

Synthesis of 3b. Inside the glovebox, complex **2b** (100 mg, 0.13 mmol) was dissolved in 10 mL of CH_2Cl_2 and combined with $AgPF_6$ (33 mg, 0.13 mmol) and 3-(3-pyridyl)coumarin (**C-1**) (30 mg, 0.13 mmol), which turned into a cloudy orange suspension. The mixture was stirred overnight at room temperature, and then filtered and the solvent was removed under vacuum. The crude product was purified by alumina column chromatography using CH_2Cl_2/ethyl acetate (1:1) and crystallization from CH_2Cl_2/Et_2O to obtain the final product as orange powder. (Yield: 88 mg, 62%). ^{1}H NMR (500 MHz, CD_3CN): δ = 9.31 (d, 1H, J = 6.5 Hz), 9.05 (d, 2H, J = 9.0 Hz), 8.93 (d, 1H, J = 8.5 Hz), 8.73 (s, 1H), 8.29–8.37 (m, 3H), 8.24–8.26 (m, 1H), 8.12–8.17 (m, 2H), 7.80–7.97 (m, 5H), 7.74–7.76 (m, 2H), 7.62–7.66 (m, 2H), 7.49–7.52 (m, 1H), 7.37–7.39 (m, 2H), 7.21 (q, 2H, J = 7.5 Hz), 7.09 (t, 3H, J = 7.5 Hz), 6.99 (t, 1H, J = 7.5 Hz), 6.83 (t, 1H, J = 7.5 Hz), 6.39 (d, 1H, J = 7.0 Hz), 6.27 (d, 1H, J = 7.5 Hz), 2.07 (s, 6H). ^{19}F NMR (470 MHz, CD_3CN): δ = –72.84 (d, 6F, J = 705.0 Hz, PF_6). $^{13}C\{^1H\}$ NMR (151 MHz, $CDCl_3$): δ = 169.1, 168.4, 165.1, 153.8, 146.5, 145.3, 145.1, 143.2, 141.7, 138.7, 137.4, 137.3, 135.6, 135.5, 135.4, 134.0, 132.7, 132.3, 132.1, 132.0, 131.4, 131.0, 130.9, 130.4, 130.3, 129.6, 129.53, 129.49, 128.98, 128.97, 128.2, 128.1, 128.04, 127.95, 127.82, 127.81, 127.3, 127.2, 126.9, 126.7, 126.6, 125.3, 124.1, 123.5, 122.8, 122.6, 122.4, 119.4, 116.3, 18.4. HRMS: *m/z* calculated for $C_{53}H_{38}F_6IrN_4O_2P$ $[M-PF_6]^+$: 955.2619, found: 955.2616.

Synthesis of 3c. A mixture of **2c** (30 mg, 0.035 mmol), 3-(3-pyridyl)coumarin (**C-1**) (7.7 mg, 0.035 mmol), and $AgPF_6$ (8.7 mg, 0.035 mmol) was dissolved in 15 mL of CH_2Cl_2 inside the glovebox. The reaction mixture was stirred for 72 h at room temperature. The AgCl was filtered off and the solvent was removed under vacuum. The orange solid was purified by washing three times with hexane and precipitation from DCM/hexane. The orange solid was then washed three times with toluene and precipitated from chloroform/pentane. Yield: 8 mg (20%) ^{1}H NMR (500 MHz, $CDCl_3$): δ = 9.26 (t, 1H, J = 4.8 Hz), 8.95 (d, 1H, J = 8.5 Hz), 8.85 (d, 1H, J = 8.0 Hz), 8.61–8.67 (m, 3H), 8.40 (d, 1H, J = 8.0 Hz), 8.08–8.14 (m, 2H), 8.03 (d, 1H, J = 8.0 Hz), 7.95–8.02 (m, 2H), 7.90 (q, 2H, J = 7.0 Hz), 7.83–7.86 (m, 3H), 7.69 (t, 3H, J = 8.3 Hz), 7.63 (t, 1H, J = 7.5 Hz), 7.52 (t, 1H, J = 7.8 Hz), 7.32 (q, 2H, J = 8.0 Hz), 7.23–7.25 (m, 1H), 7.07–7.13 (m, 5H), 7.01 (d, 1H, J = 8.0 Hz), 6.87–6.94 (m, 4H), 6.37 (d, 1H, J = 7.5 Hz), 1.89 (s, 6H). ^{19}F NMR (376 MHz, CDCl3): δ = –72.77 (d, 6F, J = 704.2 Hz, PF_6). $^{13}C\{^1H\}$ NMR (151 MHz, $CDCl_3$): δ = 169.4, 153.6, 142.7, 134.2, 134.13, 134.07, 133.5, 133.34, 133.30, 132.99, 132.96, 132.9, 132.7, 132.6, 132.5, 132.3, 130.42, 130.38, 130.3, 129.9, 129.8, 129.64, 129.56, 129.5, 129.42, 129.41, 129.37, 129.3, 129.0, 128.64,

128.58, 128.5, 128.4, 128.2, 128.1, 128.0, 127.9, 127.7, 127.19, 127.16, 127.13, 127.11, 125.54, 125.52, 125.2, 125.14, 125.12, 124.2, 123.7, 123.44, 123.35, 123.1, 122.8, 122.7, 122.6, 119.2, 116.1, 18.3. HRMS: *m/z* calculated for $C_{61}H_{42}F_6IrN_4O_2P$ $[M–C_{14}H_9O_2NPF_6]^+$: 832.2298, found: 832.2296.

Synthesis of 4a. Inside the glovebox, complex **2a** (100 mg, 0.14 mmol) was dissolved in CH_2Cl_2 (10 mL) and $AgPF_6$ (34 mg, 0.14 mmol) and **C-2** (36 mg, 0.14 mmol) were added. The mixture was stirred at room temperature for two days. The reaction was then filtered, and CH_2Cl_2 was removed under vacuum. The final product was obtained after silica gel column chromatography using CH_2Cl_2/Ethyl acetate (7:3) as the eluent and recrystallization from CH_2Cl_2/Et_2O. (Yield: 57 mg, 38%) ^1H NMR (600 MHz, CD_3CN): δ = 9.36 (d, 1H, J = 5.4 Hz), 8.91 (s, 1H), 8.72 (s, 2H), 8.44 (d, 1H, J = 8.4 Hz), 8.35 (t, 2H, J = 5.7 Hz), 8.07–8.13 (m, 2H), 7.76–7.81 (m, 2H), 7.39–7.42 (m, 6H), 7.26 (t, 1H, J = 7.8 Hz), 7.14 (d, 2H, J = 7.8 Hz), 6.64–6.73 (m, 2H), 5.83 (dd, 1H, J = 2.1 Hz), 5.73 (dd, 1H, J = 2.1 Hz), 2.11 (s, 6H). ^{19}F NMR (564 MHz, CD_3CN): δ = –72.81 (d, 6F, J = 701.6 Hz, PF_6), –106.68 (q, 1F, J = 10.2 Hz, F_2ppy), –108.01 (q, 1F, J = 10.2 Hz, J = 10.2 Hz, F_2ppy), –108.70 (t, 1F, J = 13.3 Hz, F_2ppy), –110.10 (t, 1F, J = 12.7 Hz, F_2ppy). $^{13}C\{^1H\}$ NMR (151 MHz, $CDCl_3$): δ = δ = 165.8, 165.1, 164.8 (dd, J_{CF} = 245 Hz, 4.5 Hz), 164.24, 164.0, 163.4 (dd, J_{CF} = 229 Hz, 11.3 Hz), 162.6 (dd, J_{CF} = 221 Hz, 11.3 Hz), 161.6 (dd, J_{CF} = 246 Hz, 12 Hz), 159.4, 158.9, 156.7, 155.5, 153.7, 152.8, 151.8, 150.0, 140.0, 139.8, 139.7, 136.0, 135.8, 135.7, 135.5, 131.3, 130.7, 130.1, 128.3, 126.4, 125.6, 124.4, 124.3, 124.2, 120.7, 117.9, 117.3, 116.7, 115.7, 114.9, 114.2, 114.1, 113.0, 112.8, 101.1 (t, J_{CF} = 30.0 Hz), 99.6 (t, J_{CF} = 28.3 Hz), 18.4. HRMS: *m/z* calculated for $C_{46}H_{30}F_{10}IrN_4O_4P$ $[M−PF_6]^+$: 971.1827, found: 971.1819.

Synthesis of 4b. Inside the glovebox, complex **2b** (120 mg, 0.16 mmol) was dissolved in 10 mL DCM and combined with $AgPF_6$ (40 mg, 0.16 mmol) and an excess amount of **C-2** (50 mg, 0.19 mmol). The mixture was allowed to react at room temperature for 2 days. The reaction mixture was then filtered and the solvent was removed under vacuum. The crude product was purified through alumina column chromatography using CH_2Cl_2/ethyl acetate (4:1) and crystallization from CH_2Cl_2/Et_2O, obtaining the final product as orange powder. (Yield: 65 mg, 36%) ^1H NMR (600 MHz, CD_3CN): δ = 9.28 (d, 1H, J = 6.6 Hz), 9.04 (d, 1H, J = 8.4 Hz), 8.93 (d, 1H, J = 8.4 Hz), 8.88 (s, 1H), 8.78 (s, 2H), 8.34 (d, 1H, J = 8.4 Hz), 8.29 (d, 1H, J = 7.8 Hz), 8.26 (d, 1H, J = 6.0 Hz), 8.13–8.17 (m, 2H), 7.74–7.90 (m, 8H), 7.38–7.43 (m, 4H), 7.21 (t, 1H, J = 7.5 Hz), 7.14–7.15 (m, 1H), 7.07–7.10 (m, 3H), 6.97 (t, 1H, J = 7.0 Hz), 6.83 (t, 1H, J = 7.8 Hz), 6.40 (d, 1H, J = 7.8 Hz), 6.24 (d, 1H, J = 7.8 Hz), 2.06 (s, 6H). ^{19}F NMR (564 MHz, CD_3CN): δ = –72.51 (d, 6F, J = 695.4 Hz, PF_6). $^{13}C\{^1H\}$ NMR (151 MHz, $CDCl_3$): δ = 169.2, 168.9, 165.8, 165.3, 155.3, 153.9, 149.4, 146.4, 146.2, 145.0, 144.8, 144.1, 141.1, 137.24, 137.17, 135.3, 134.7, 132.5, 132.24, 132.20, 132.1, 131.3, 130.9, 130.8, 130.4, 130.3, 130.0, 129.8, 129.6, 129.1, 128.6, 128.3, 128.0, 127.92, 127.85, 127.7, 127.4, 127.03, 126.99, 126.7, 126.61, 126.57, 125.1, 124.0, 123.1, 122.7, 121.9, 118.0, 116.9, 115.4, 18.5. HRMS: *m/z* calculated for $C_{61}H_{42}F_6IrN_4O_2P$ $[M–C_{15}H_9O_4NPF_6]^+$: 732.1985, found: 732.1984.

Synthesis of 5a. A mixture of **1a** (41 mg, 0.034 mmol) and $AgPF_6$ (17 mg, 0.068 mmol) was dissolved in 15 mL of DCM in the glovebox. After stirring for 2 hours, 3-(3-pyridyl)coumarin (**C-1**) (30 mg, 0.136 mmol) was added and the reaction mixture was stirred for 48 h at room temperature. The AgCl was filtered off and the solvent was removed under vacuum. The yellow solid was then washed three times with diethyl ether and purified by precipitation from CH_2Cl_2/diethyl ether, followed by washing with toluene three times and recrystallization from CH_2Cl_2/hexane and chloroform/hexane/diethyl ether. (Yield: 25 mg, 32%). ^1H NMR (400 MHz, $CDCl_3$): δ = 8.87 (s, 2H), 8.78 (d, 2H, J = 5.6 Hz), 8.46 (d, 2H, J = 5.6 Hz), 8.23 (d, 2H, J = 8.4 Hz), 8.07 (d, 2H, J = 8.0 Hz), 7.89 (t, 4H, J = 8.4 Hz), 7.50–7.60 (m, 8H), 7.26–7.31 (m, 4H), 6.43–6.49 (m, 2H), 5.80–5.83 (dd, 2H, J = 2.0 Hz). ^{19}F NMR (564 MHz, CD_3CN): δ = –72.91 (d, 6F, J = 679.6 Hz, PF_6), –107.61 (q, 2F, J = 10.3 Hz, F_2ppy), –110.18 (t, 2F, J = 13.0 Hz, F_2ppy). $^{13}C\{^1H\}$ NMR (151 MHz, $CDCl_3$): δ = 166.6, 164.3, 163.5 (dd, J_{CF} = 274 Hz, 9.8 Hz), 161.0 (dd, J_{CF} = 211 Hz, 12 Hz), 153.9, 151.5, 151.1, 149.7, 143.3, 139.4, 138.6, 133.9, 132.8, 129.2, 128.1, 127.2, 125.2, 124.5, 123.8, 123.6, 122.9, 119.2, 116.5, 114.5, 99.4 (t, J_{CF} = 30.0 Hz). HRMS: *m/z* calculated for $C_{50}H_{30}F_{10}IrN_4O_4P$ $[M−PF_6]^+$: 1019.1827, found: 1019.1802.

References

1 A. Maity, J. C. Kölsch, H. Na and T. S. Teets, *Dalton Trans.*, 2017, **46**, 11757–11767.
2 H. Na, M. Song and T. S. Teets, *Chem. – Eur. J.*, 2019, **25**, 4833–4842.
3 K. Suzuki, A. Kobayashi, S. Kaneko, K. Takehira, T. Yoshihara, H. Ishida, Y. Shiina, S. Oishi and S. Tobita, *Phys. Chem. Chem. Phys.*, 2009, **11**, 9850–9860.
4 F. Vögtle, M. Plevoets, M. Nieger, G. C. Azzellini, A. Credi, L. De Cola, V. De Marchis, M. Venturi and V. Balzani, *J. Am. Chem. Soc.*, 1999, **121**, 6290–6298.
5 W. Spiller, H. Kliesch, D. Wöhrle, S. Hackbarth, B. Röder and G. Schnurpfeil, *J. Porphyr. Phthalocyanines*, 1998, **02**, 145–158.
6 S. Mathai, T. A. Smith and K. P. Ghiggino, *Photochem. Photobiol. Sci.*, 2007, **6**, 995–1002.
7 O. Shimizu, J. Watanabe, K. Imakubo and S. Naito, *Chem. Lett.*, 1999, **28**, 67–68.
8 K. I. Salokhiddinov, I. M. Byteva and G. P. Gurinovich, *J. Appl. Spectrosc.*, 1981, **34**, 561–564.
9 G. M. Sheldrick, *Acta Crystallogr. Sect. C Struct. Chem.*, 2015, **71**, 3–8.
10 A. L. Spek, *Acta Crystallogr. D Biol. Crystallogr.*, 2009, **65**, 148–155.
11 R. B. Moffett, *J. Med. Chem.*, 1964, **7**, 446–449.
12 V. Garg, G. Kodis, P. A. Liddell, Y. Terazono, T. A. Moore, A. L. Moore and D. Gust, *J. Phys. Chem. B*, 2013, **117**, 11299–11308.

Table S1. Summary of X-ray crystallographic data for **3a**, **4a** and **5a**.

	**3a**·$0.5C_7H_8$	**4a**·$1.5C_6H_6$	**5a**
CCDC	2126601	2126602	2126603
Crystal data			
Chemical formula	$C_{48.50}H_{34}F_{10}IrN_4O_2P$	$C_{55}H_{39}F_{10}IrN_4O_4P$	$C_{50}H_{30}F_{10}IrN_4O_4P$
M_r	1117.96	1233.07	1163.95
Crystal system, space group	Triclinic, $P\overline{1}$	Triclinic, $P\overline{1}$	Triclinic, $P\overline{1}$
Temperature (K)	123	123	123
a, b, c (Å)	12.373 (6), 19.541 (9), 21.137(10)	12.0293 (3), 13.6426 (3), 15.8219 (3)	11.885 (2), 13.195 (3), 16.517 (3)
α, β, γ (°)	63.579 (5), 75.787 (5), 71.958 (5)	79.160 (1), 81.948 (1), 71.953 (1)	105.127 (2), 97.590 (2), 101.773 (2)
V (Å^3)	4317 (3)	2415.43 (9)	2400.2 (8)
Z	4	2	2
Radiation type	Mo $K\alpha$	Mo $K\alpha$	Mo $K\alpha$
μ (mm^{-1})	3.22	2.89	2.90
Crystal size (mm)	0.33 × 0.32 × 0.05	0.74 × 0.43 × 0.42	0.67 × 0.39 × 0.13
Data collection			
Diffractometer	Bruker *APEX*-II CCD	Bruker *APEX*-II CCD	Bruker *APEX*-II CCD
Absorption correction	Empirical (using intensity measurements) twinabs	Multi-scan *SADABS*	Empirical (using intensity measurements) *SADABS*
T_{min}, T_{max}	0.501, 0.746	0.569, 0.746	0.546, 0.746
No. of measured, independent and observed [$I > 2\sigma(I)$] reflections	16458, 16458, 14158	31358, 9804, 9473	33955, 11009, 10052
R_{int}	0.0625	0.020	0.032
(sin θ/λ)$_{max}$ (Å^{-1})	0.658	0.625	0.650
Refinement			
$R[F^2 > 2\sigma(F^2)]$, $wR(F^2)$, S	0.045, 0.120, 1.06	0.023, 0.058, 1.05	0.030, 0.075, 1.05
No. of reflections	16458	9804	11009
No. of parameters	1204	697	668
No. of restraints	134	153	274
$\Delta\rho_{max}$, $\Delta\rho_{min}$ (e Å^{-3})	2.42, -2.19	1.41, -0.94	2.82, -1.03

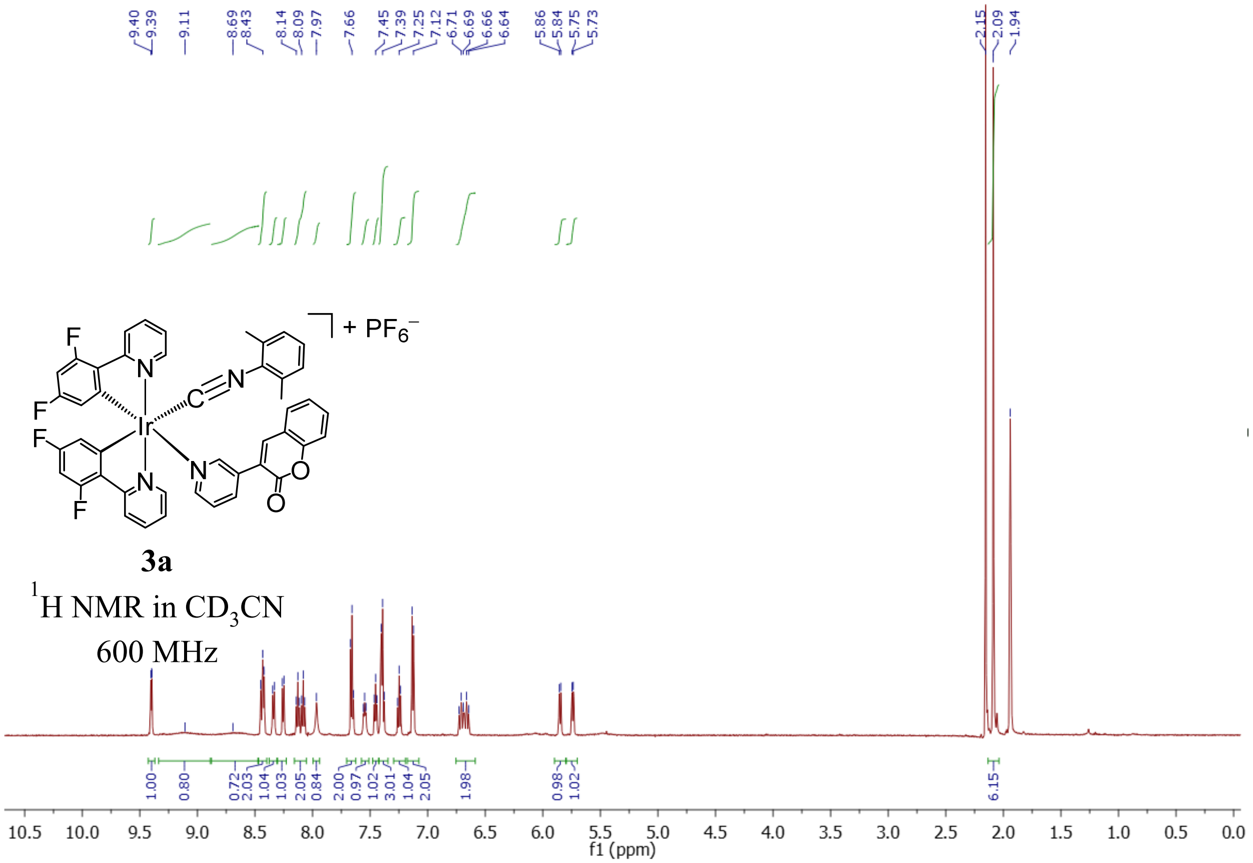

Fig. S1. ^{1}H NMR spectrum of complex **3a**, recorded at 600 MHz in CD_3CN.

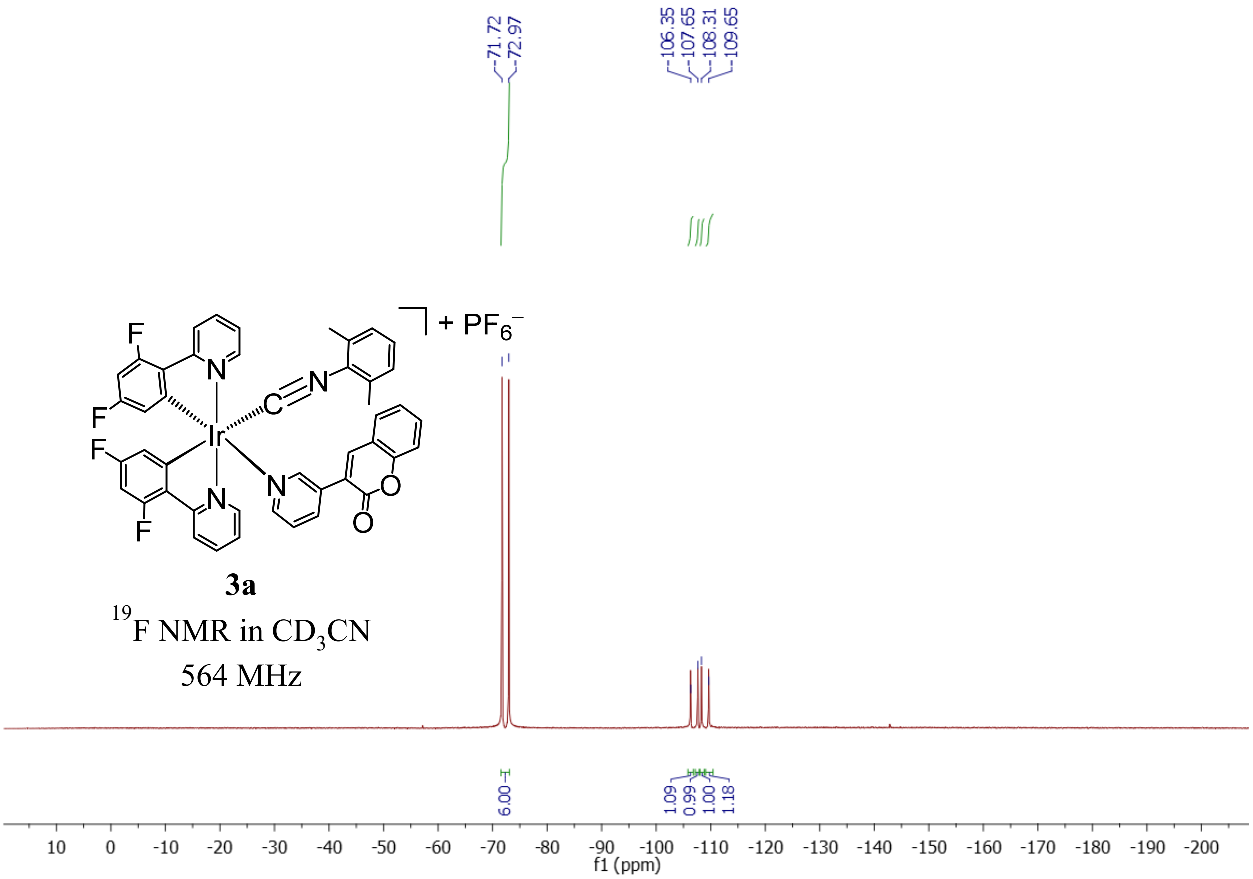

Fig. S2. ^{19}F NMR spectrum of **3a** recorded at 564 MHz in CD_3CN.

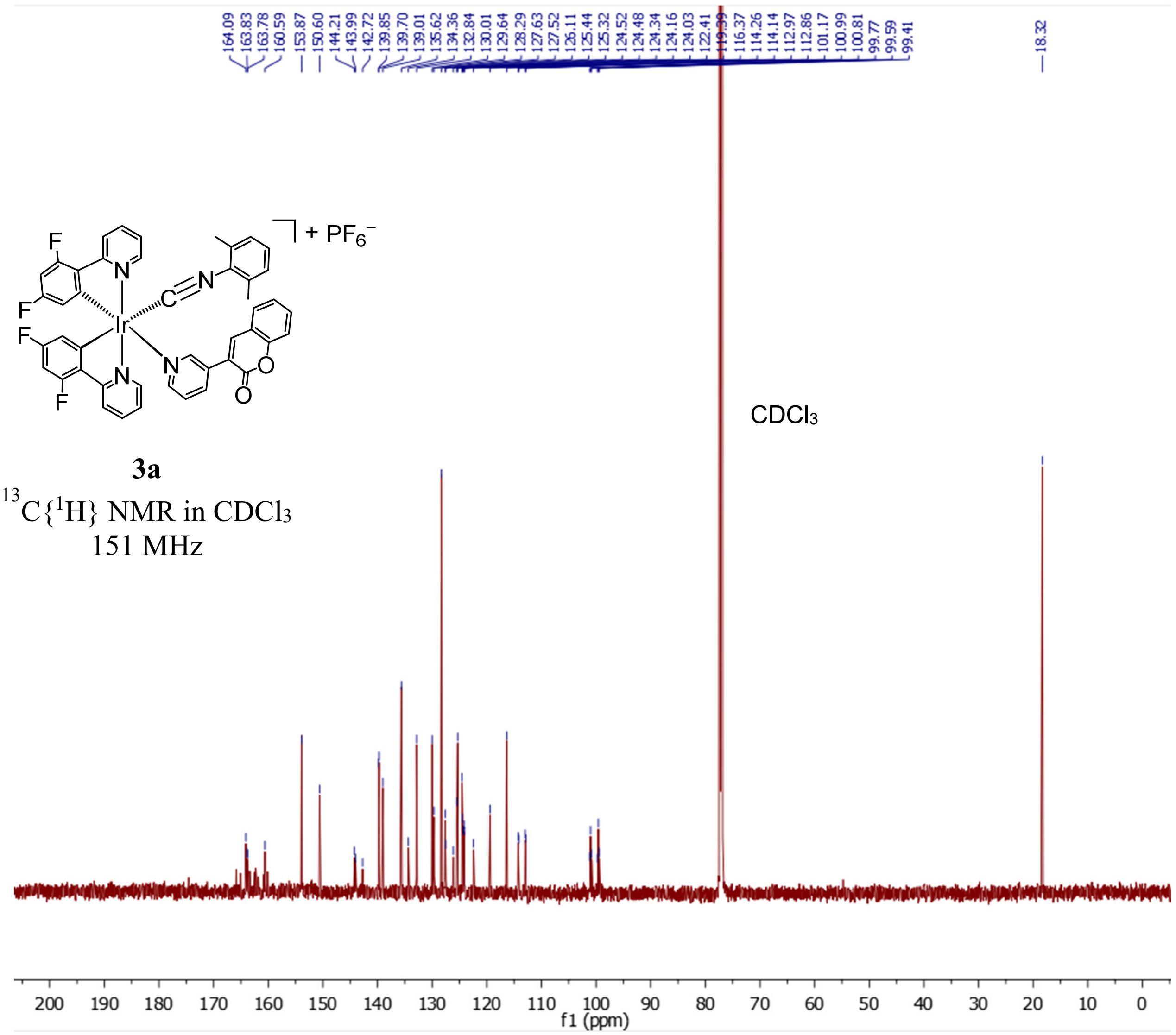

Fig. S3. $^{13}C\{^{1}H\}$ NMR spectrum of **3a** recorded at 151 MHz in $CDCl_3$.

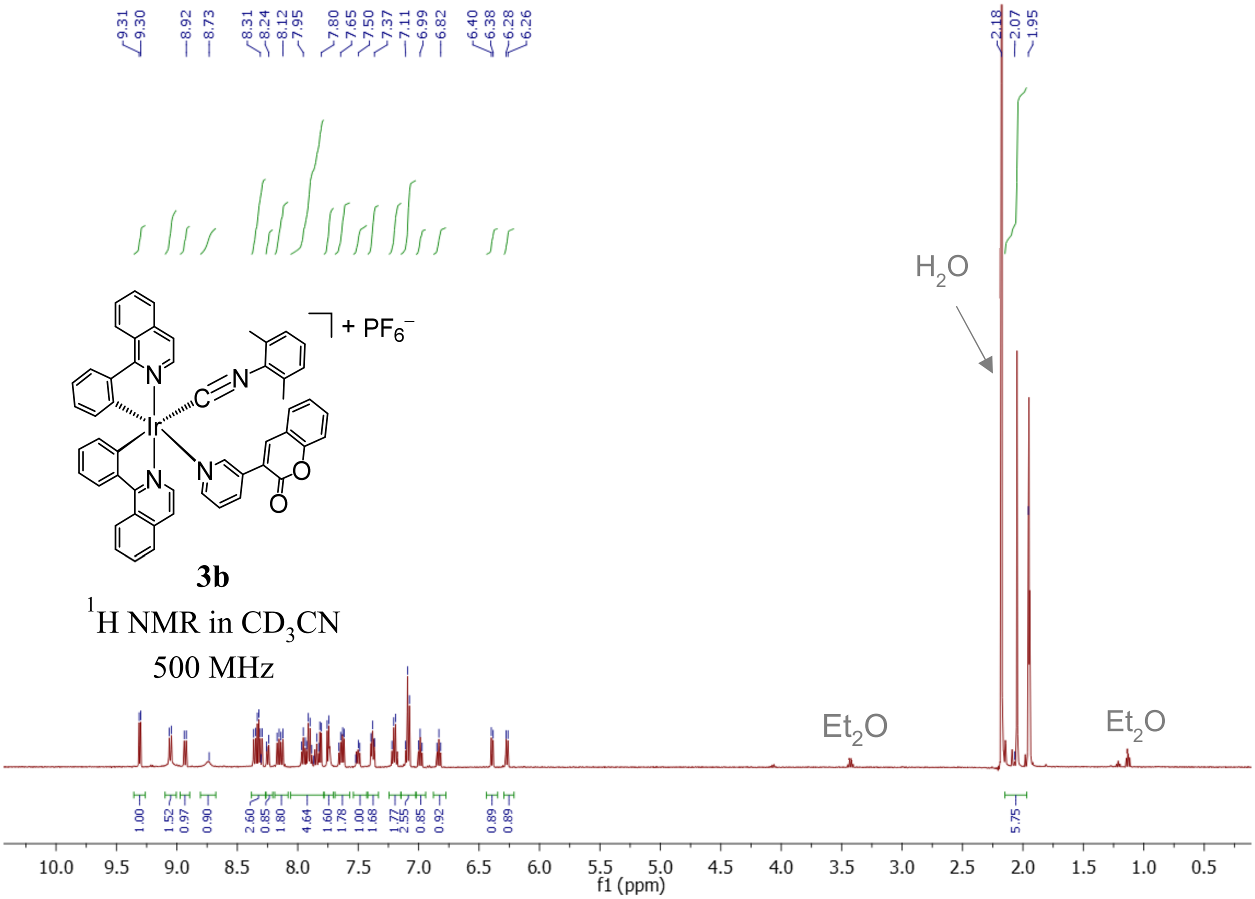

Fig. S4. ^{1}H NMR spectrum of **3b** recorded at 500 MHz in CD_3CN.

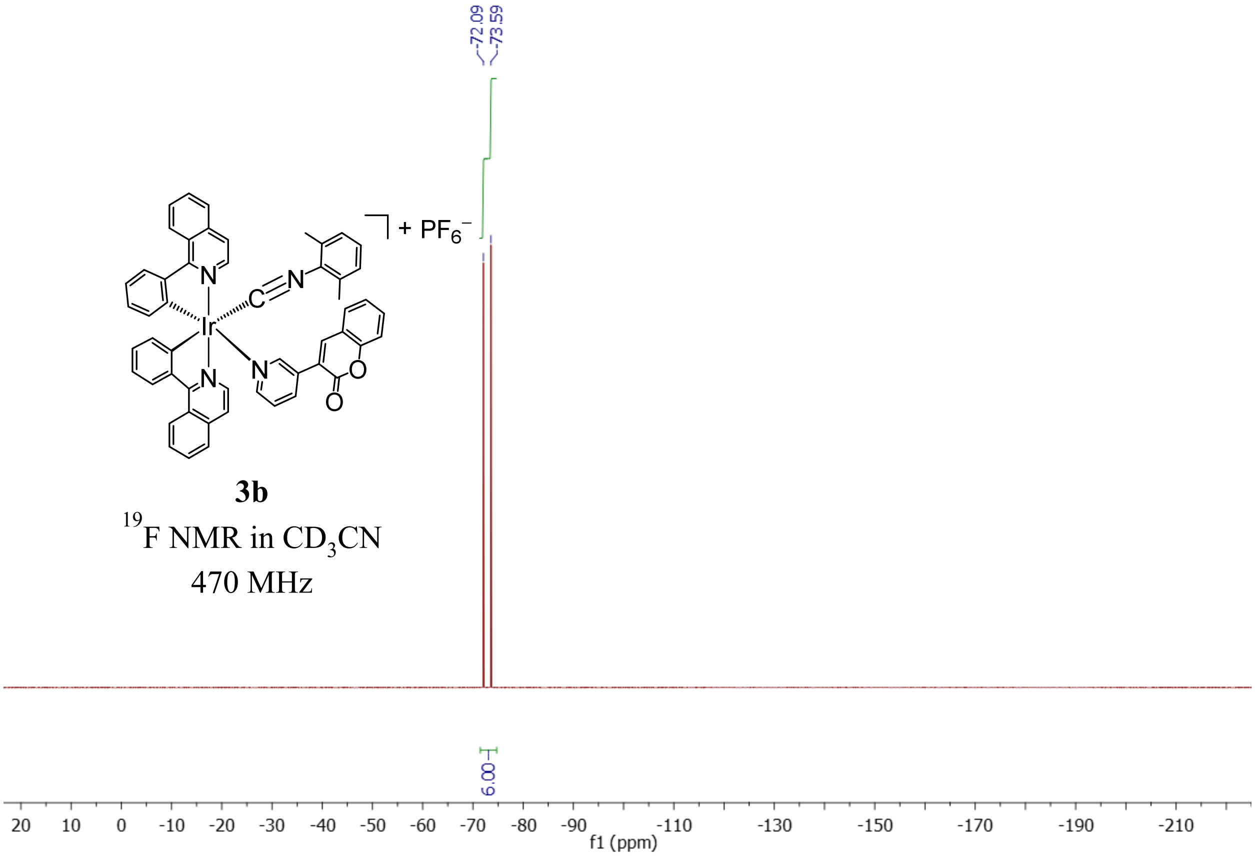

Fig. S5. ^{19}F NMR spectrum of **3b** recorded at 470 MHz in CD_3CN.

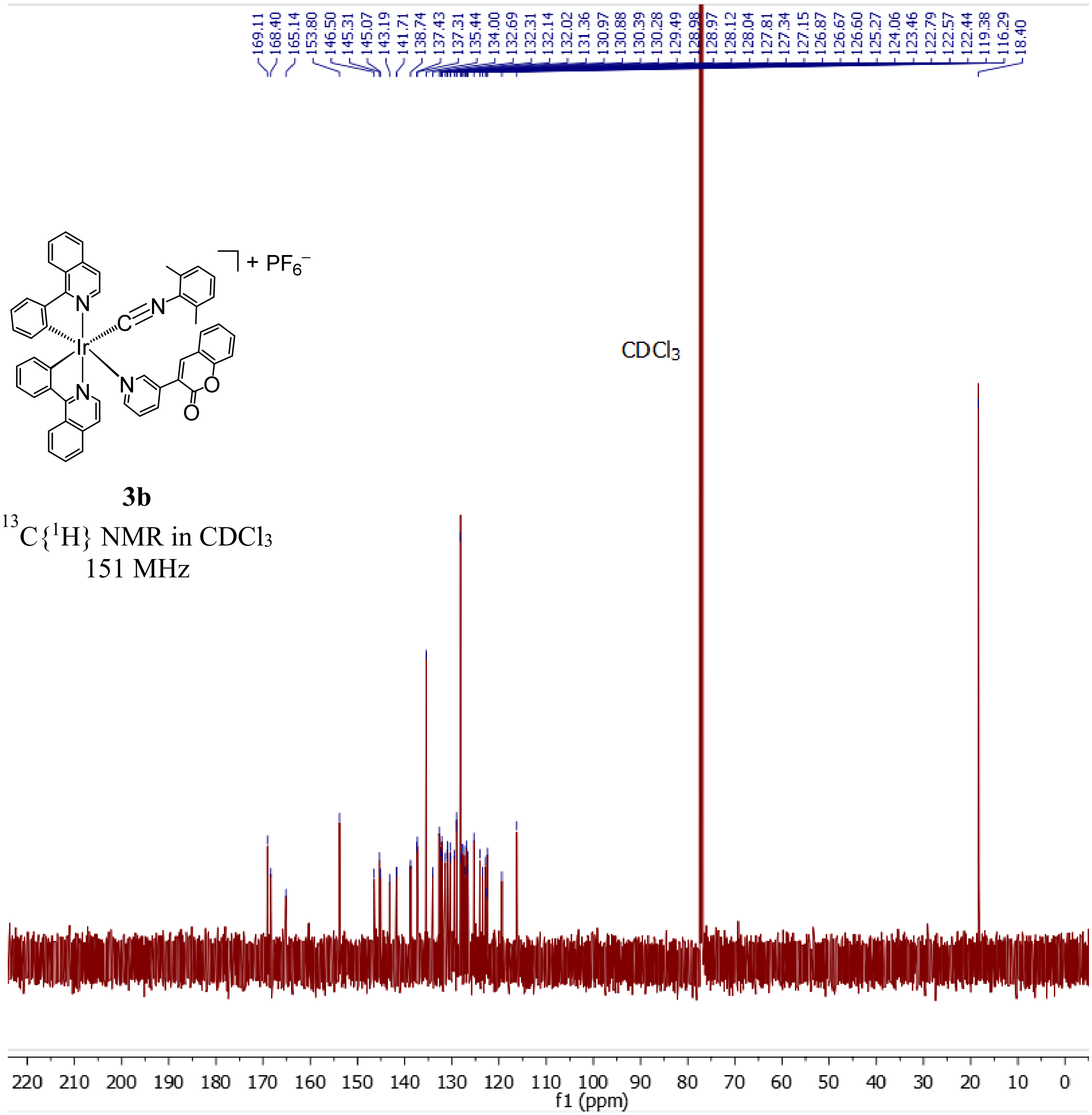

Fig. S6. $^{13}C\{^{1}H\}$ NMR spectrum of **3b** recorded at 151 MHz in $CDCl_3$.

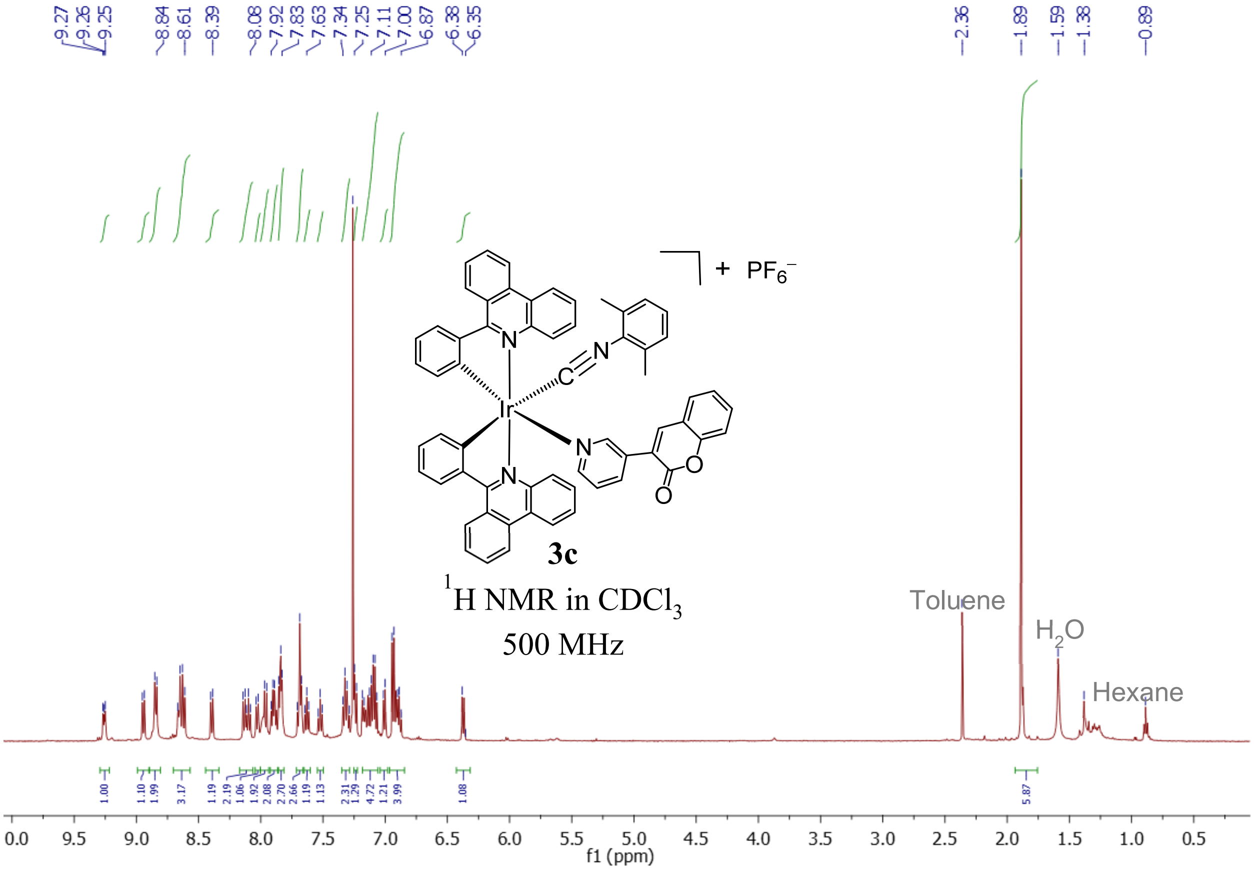

Fig. S7. ^{1}H NMR spectrum of **3c** recorded at 500 MHz in $CDCl_3$.

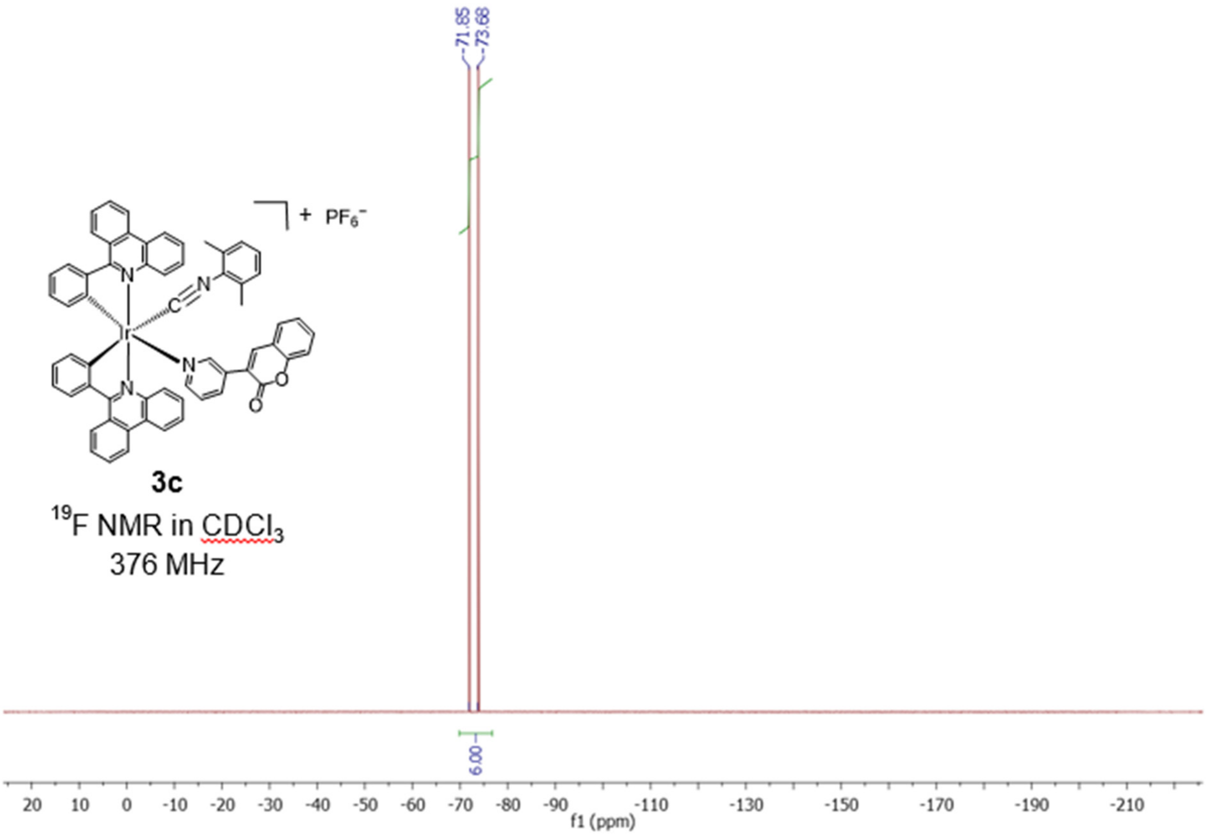

Fig. S8. ^{19}F NMR spectrum of **3c** recorded at 376 MHz in $CDCl_3$.

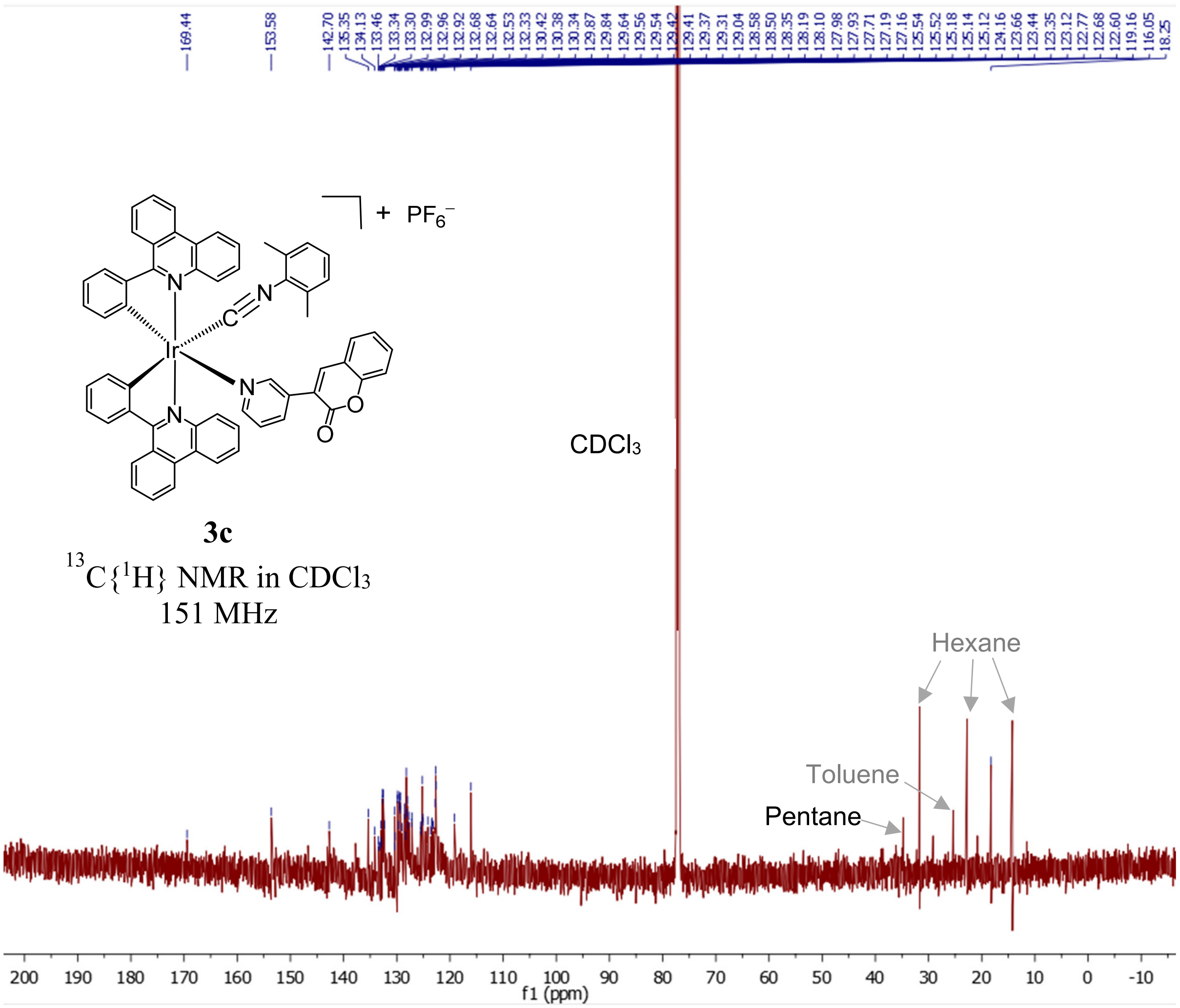

Fig. S9. $^{13}C\{^{1}H\}$ NMR spectrum of **3c** recorded at 151 MHz in $CDCl_3$.

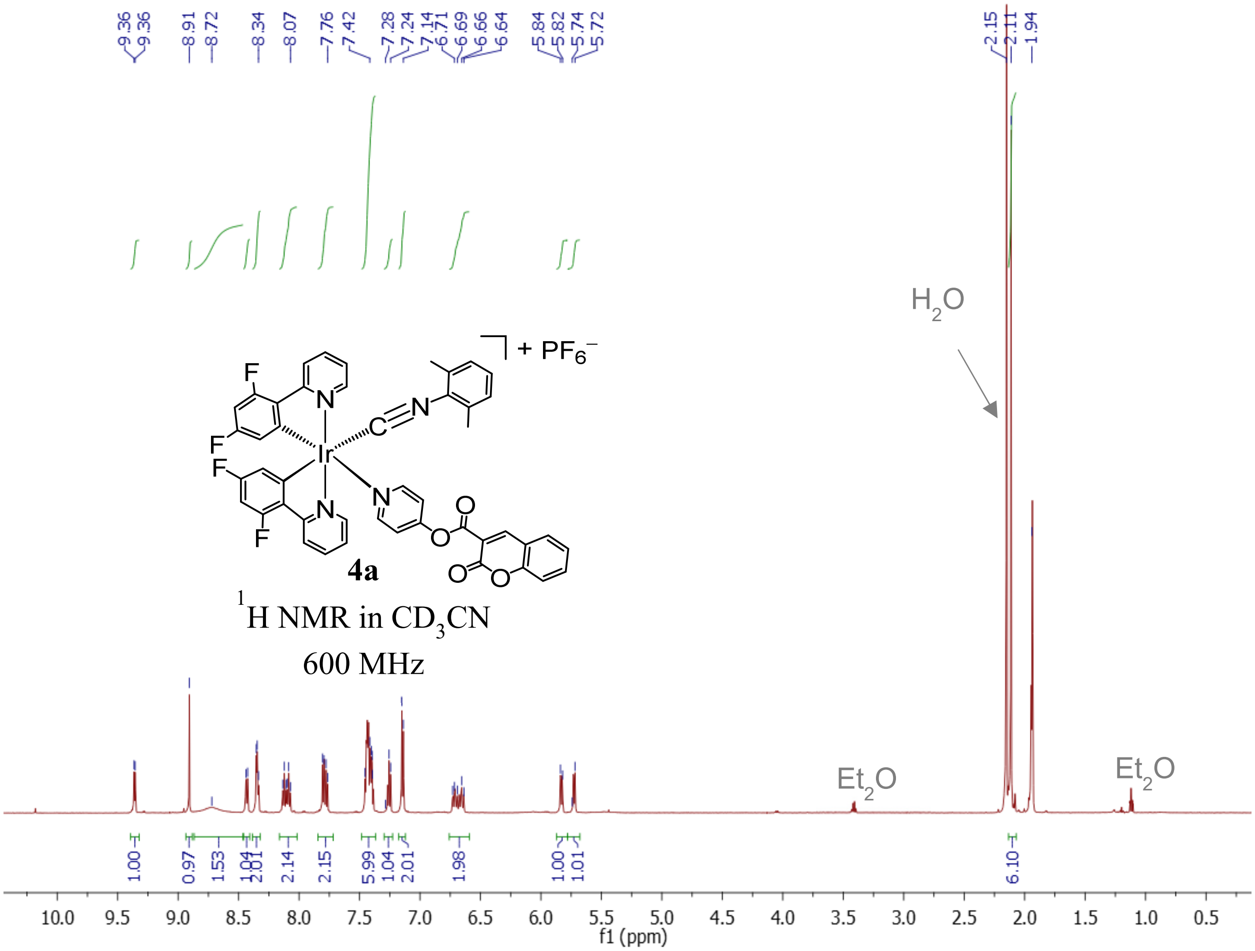

Fig. S10. ^{1}H NMR spectrum of **4a** recorded at 600 MHz in CD_3CN.

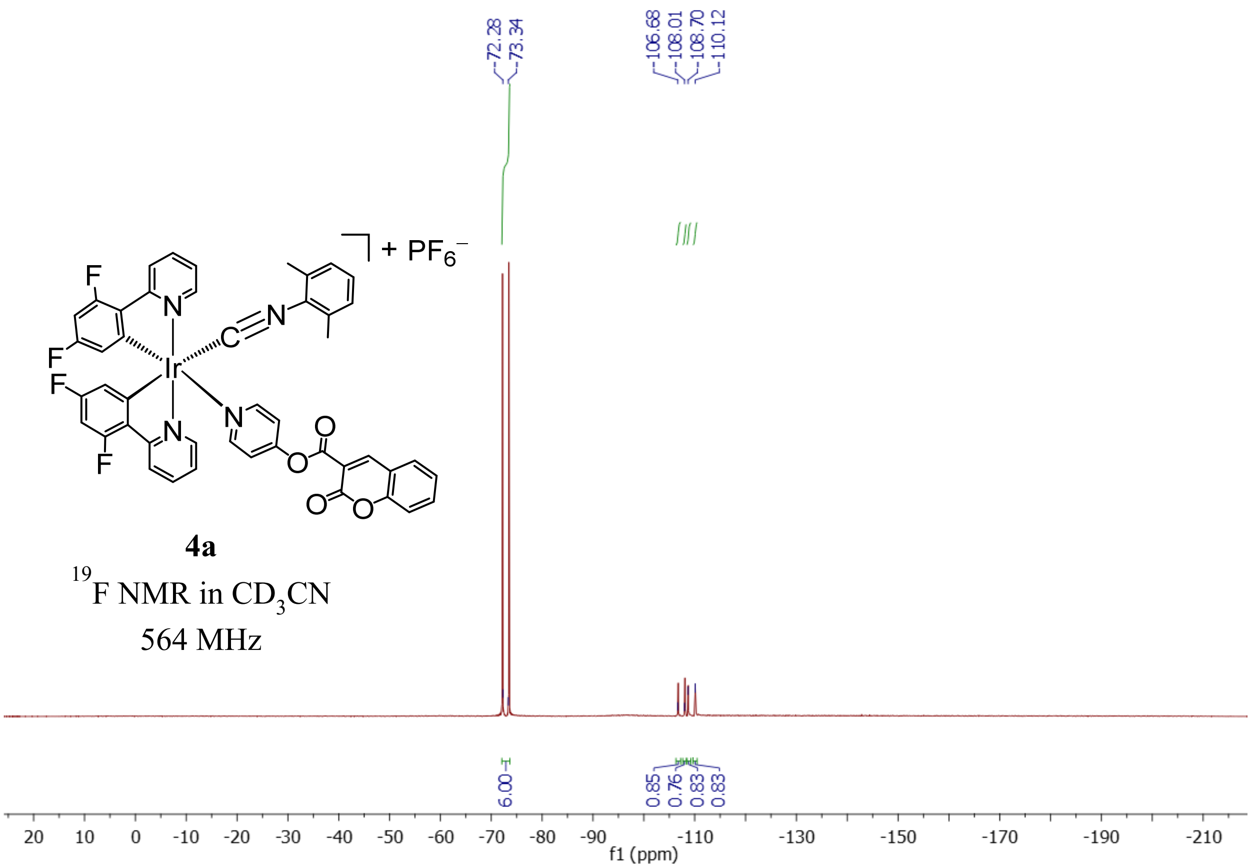

Fig. S11. ^{19}F NMR spectrum of **4a** recorded at 564 MHz in CD_3CN.

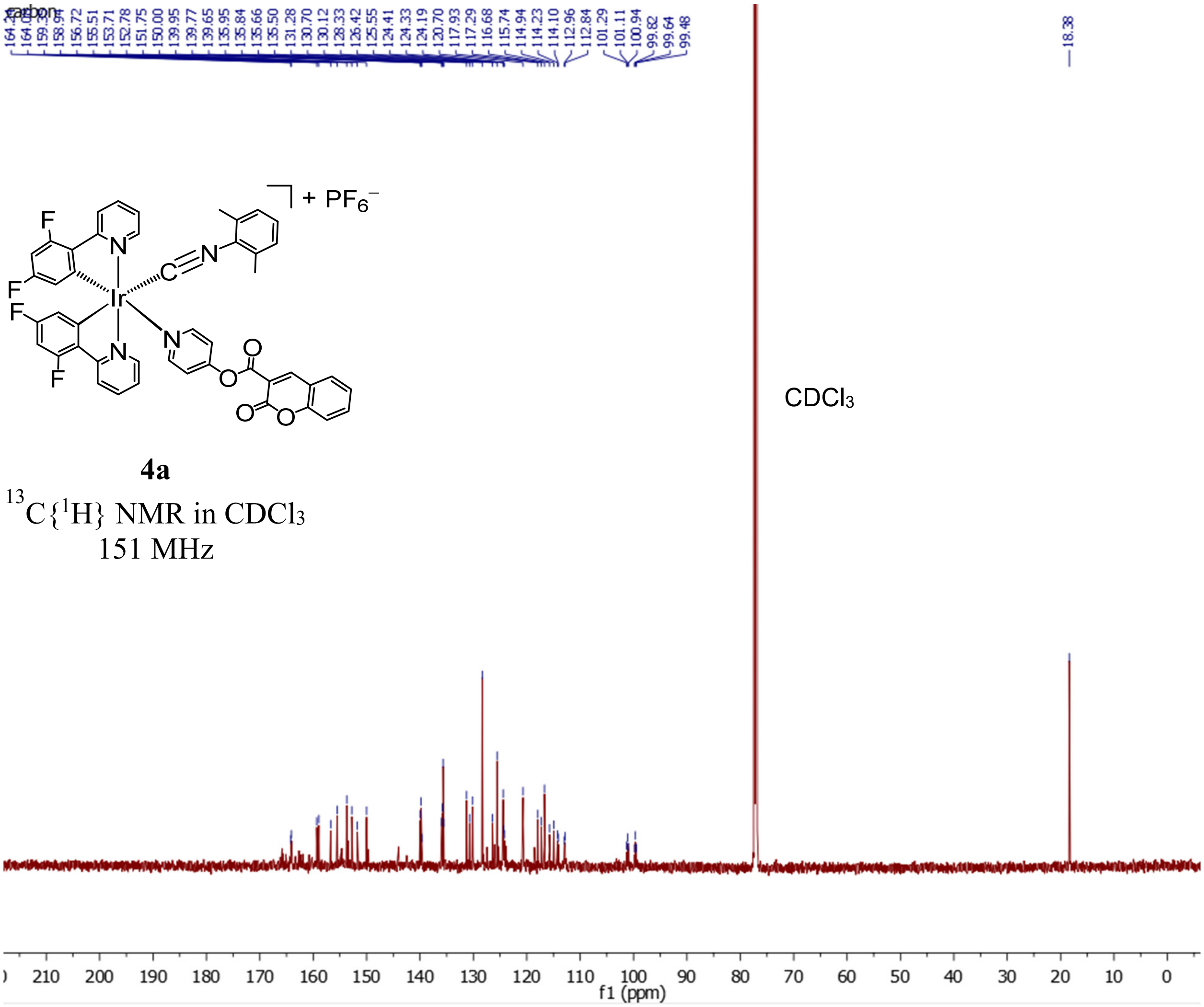

Fig. S12. $^{13}C\{^{1}H\}$ NMR spectrum of **4a** recorded at 151 MHz in $CDCl_3$.

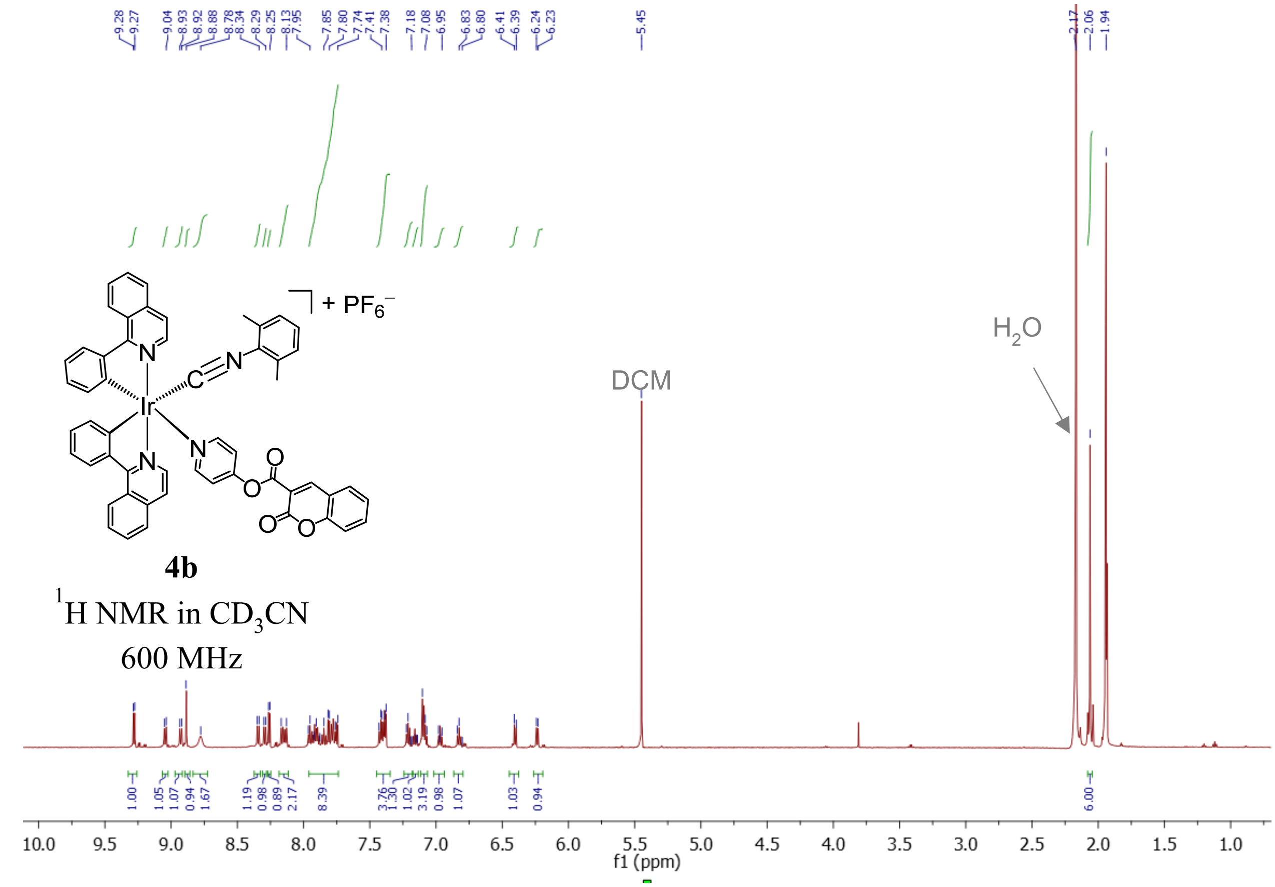

Fig. S13. ^{1}H NMR spectrum of **4b** recorded at 600 MHz in CD_3CN.

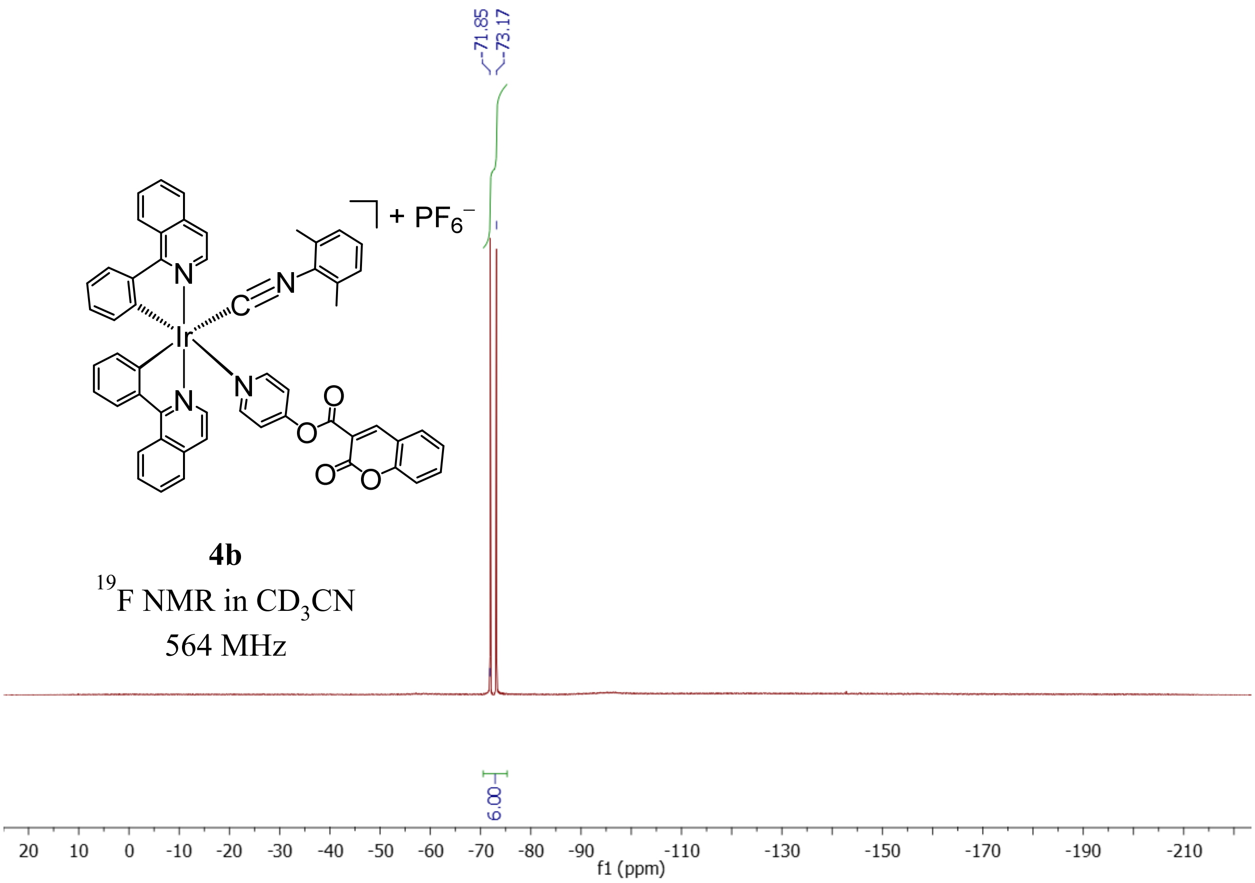

Fig. S14. ^{19}F NMR spectrum of **4b** recorded at 564 MHz in CD_3CN.

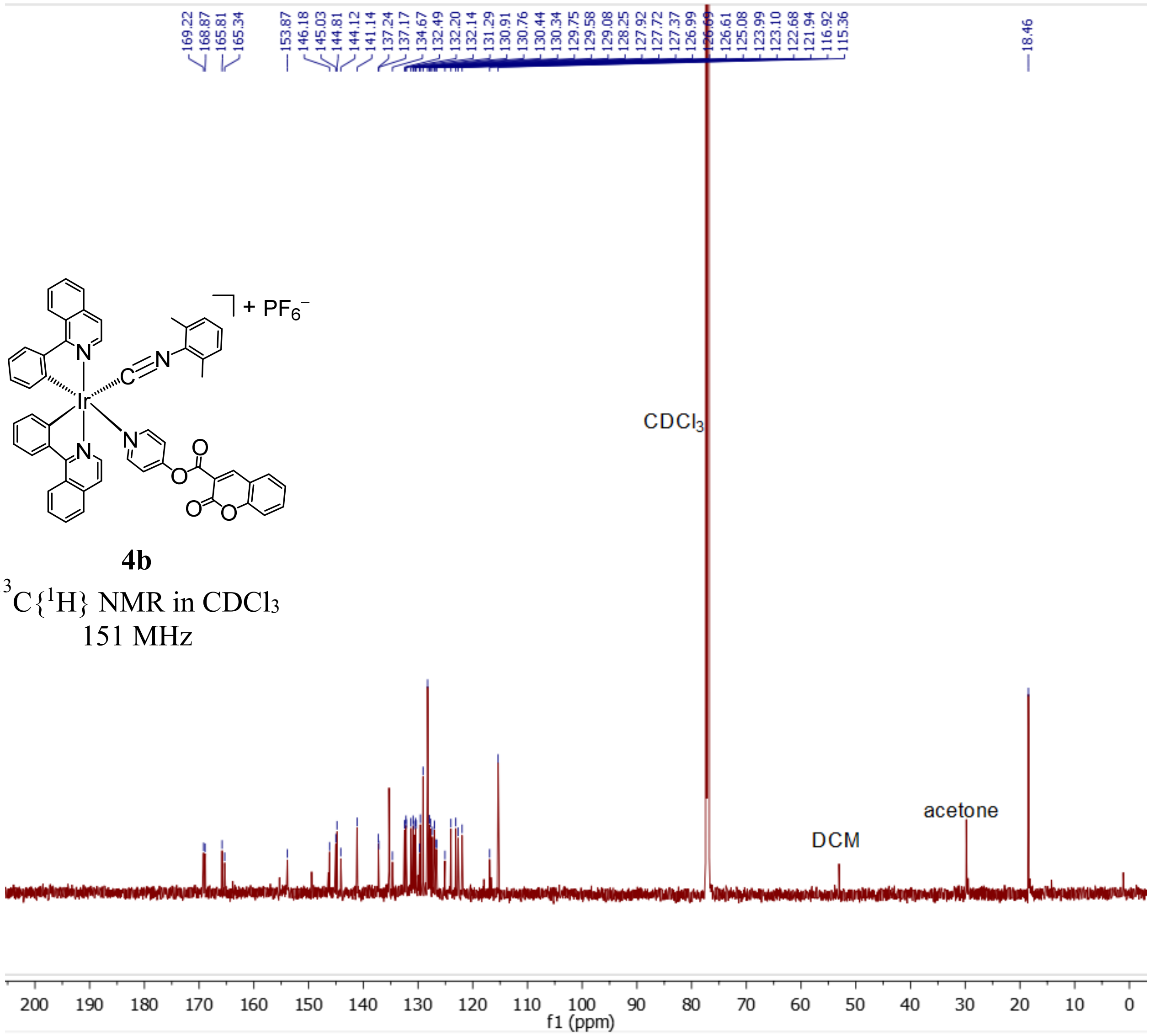

Fig. S15. $^{13}C\{^1H\}$ NMR spectrum of **4b** recorded at 151 MHz in $CDCl_3$.

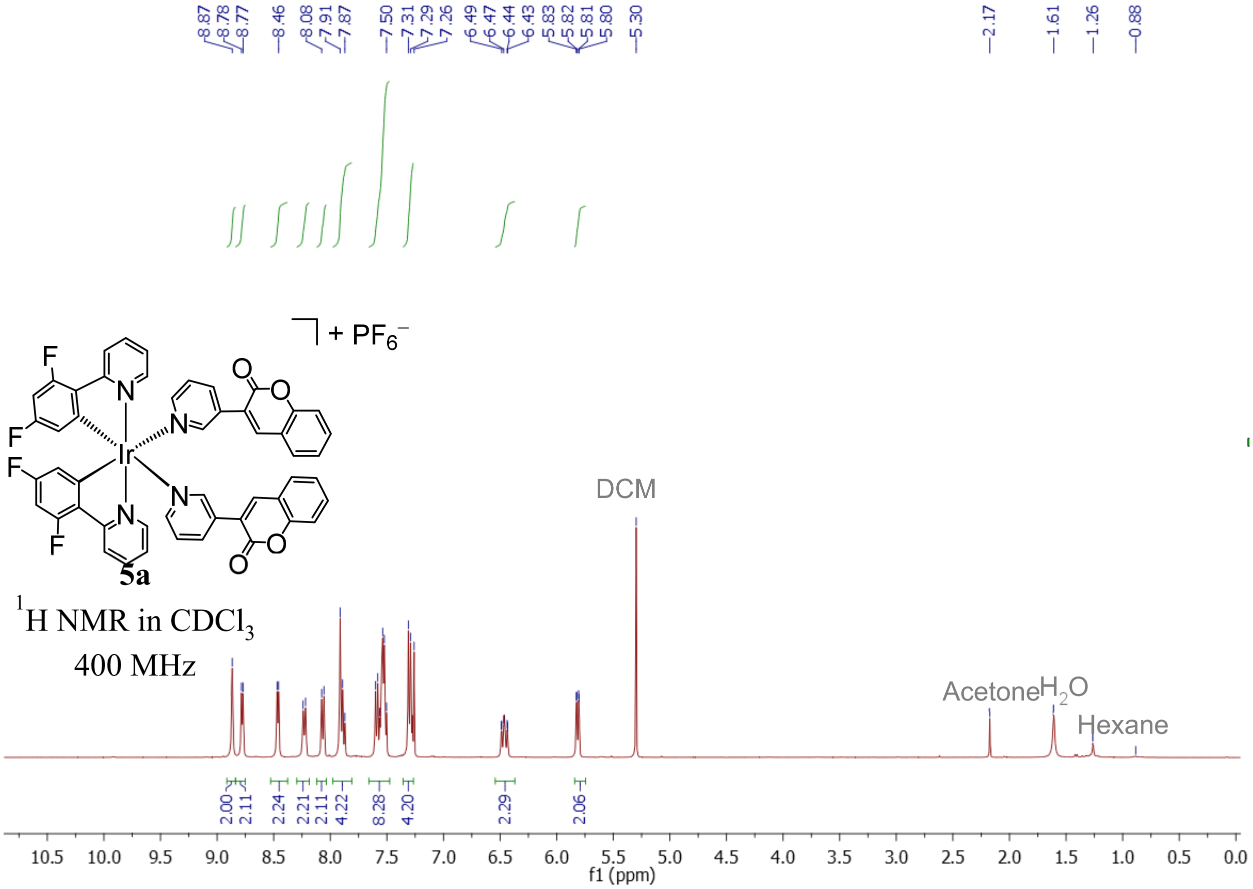

Fig. S16. ^{1}H NMR spectrum of **5a** recorded at 400 MHz in $CDCl_3$.

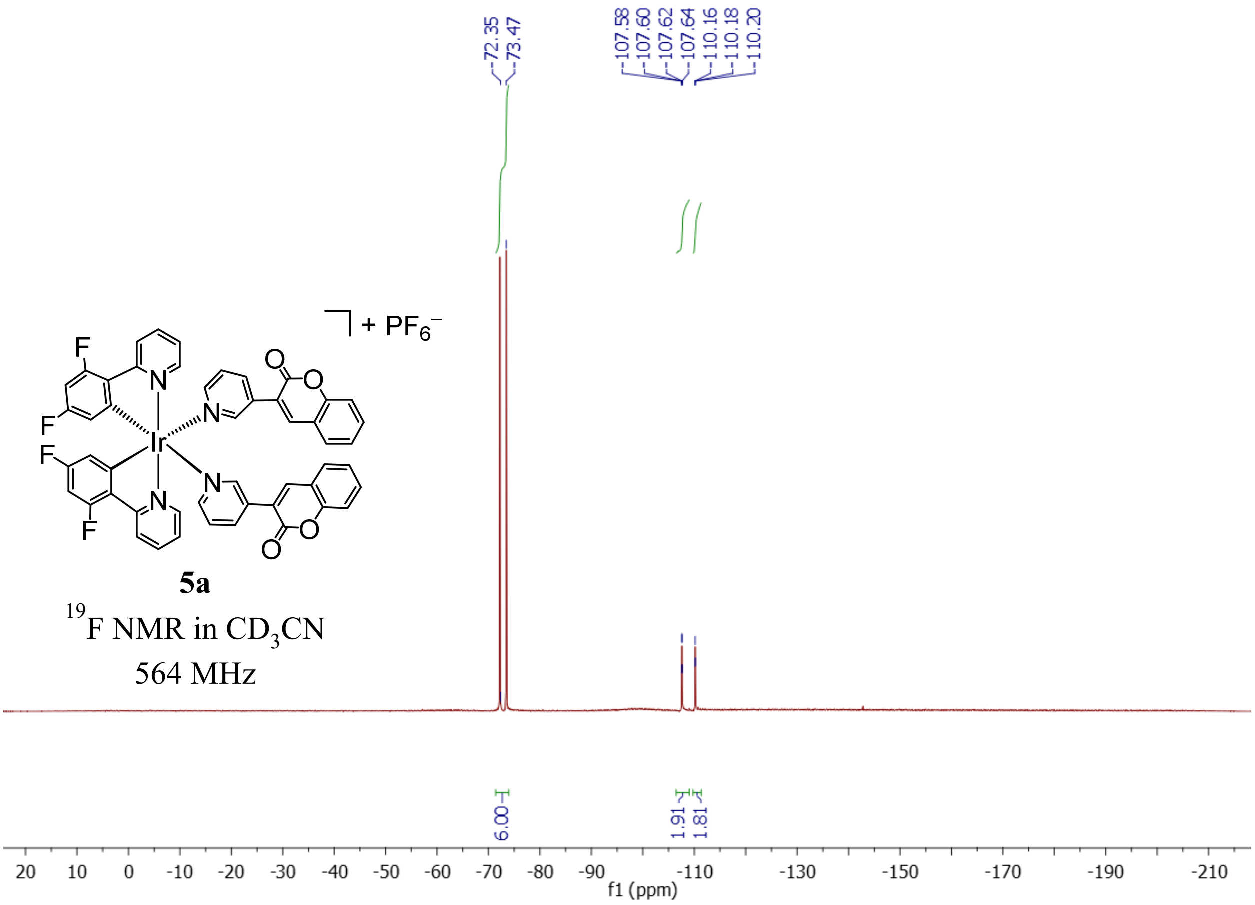

Fig. S17. ^{19}F NMR spectrum of **5a** recorded at 564 MHz in CD_3CN.

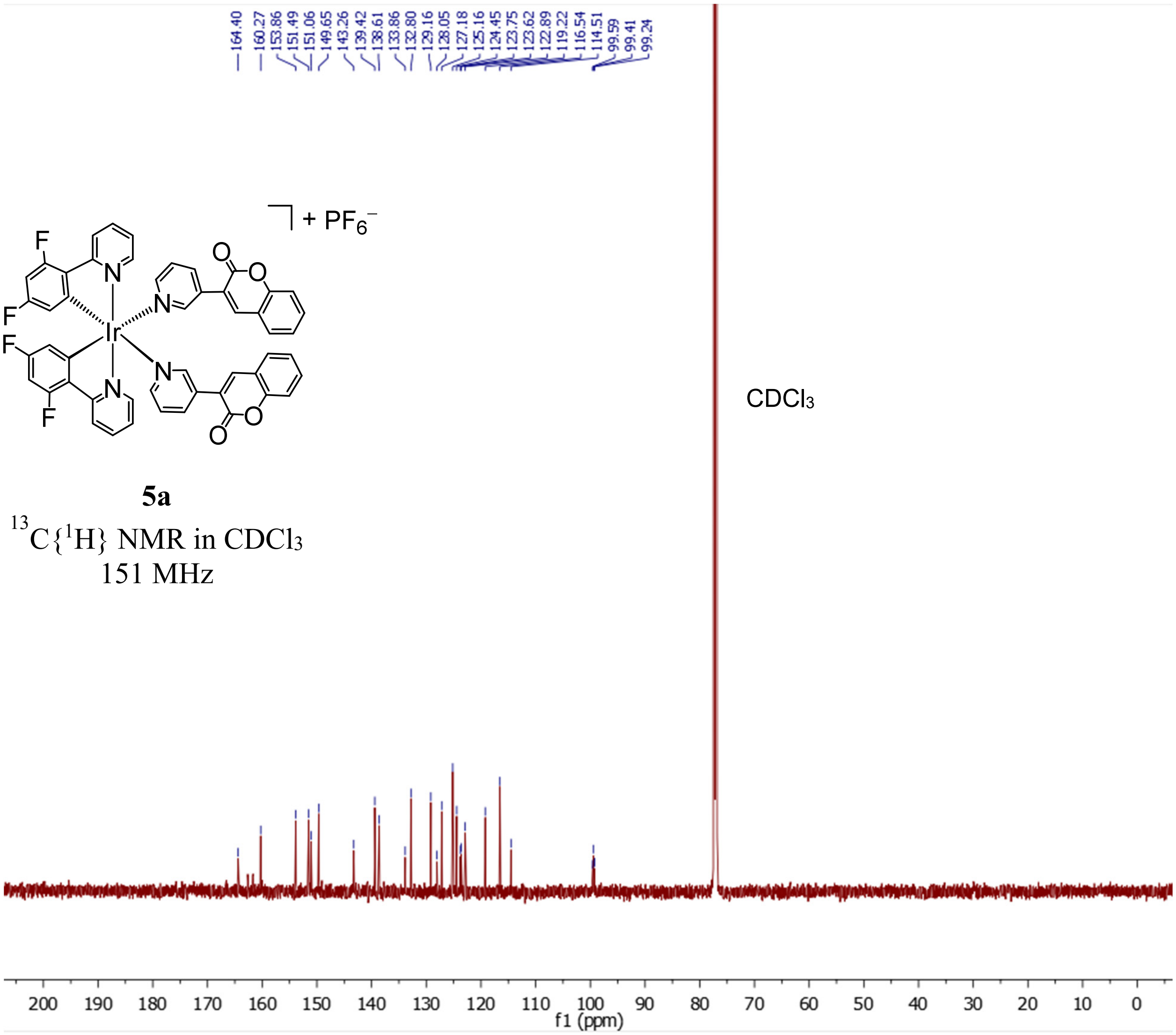

Fig. S18. $^{13}C\{^1H\}$ NMR spectrum of **5a** recorded at 151 MHz in $CDCl_3$.

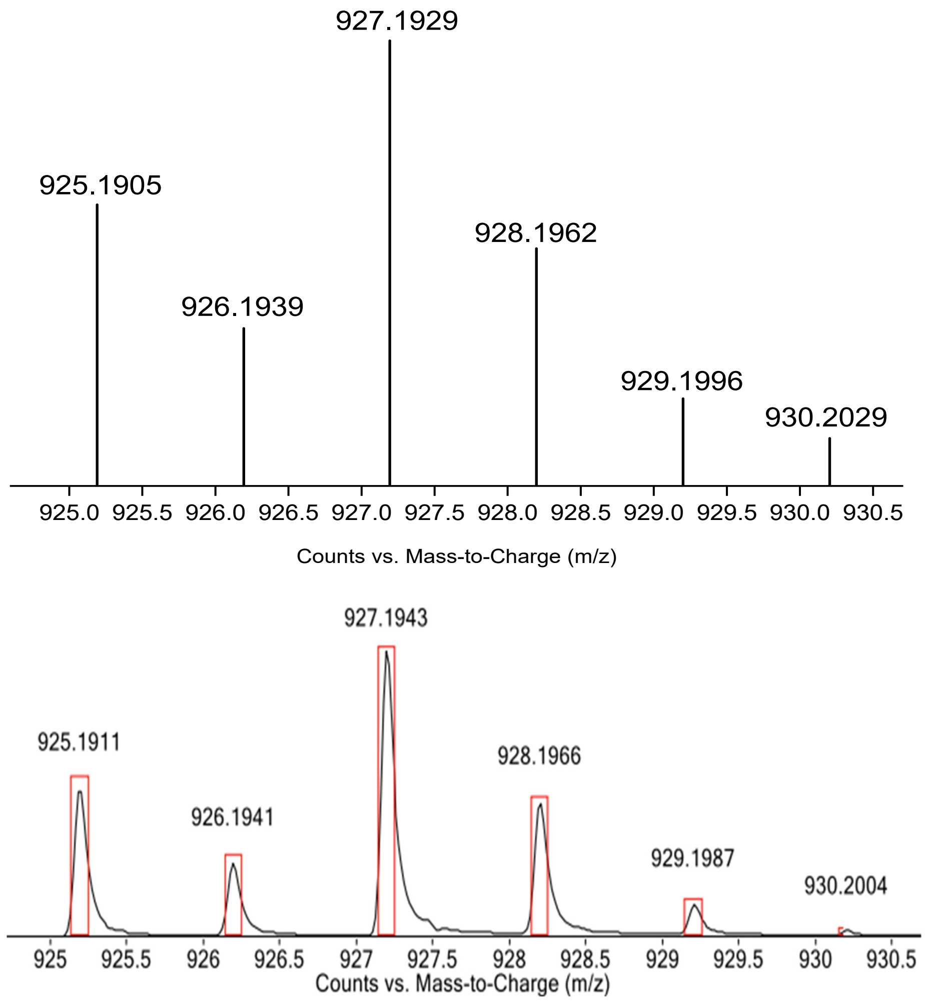

Fig. S19. Simulated (top) and experimental (bottom) ESI-MS data for complex **3a**, showing the isotropic distribution pattern for the molecular ion peak ($[M - PF_6]^+$).

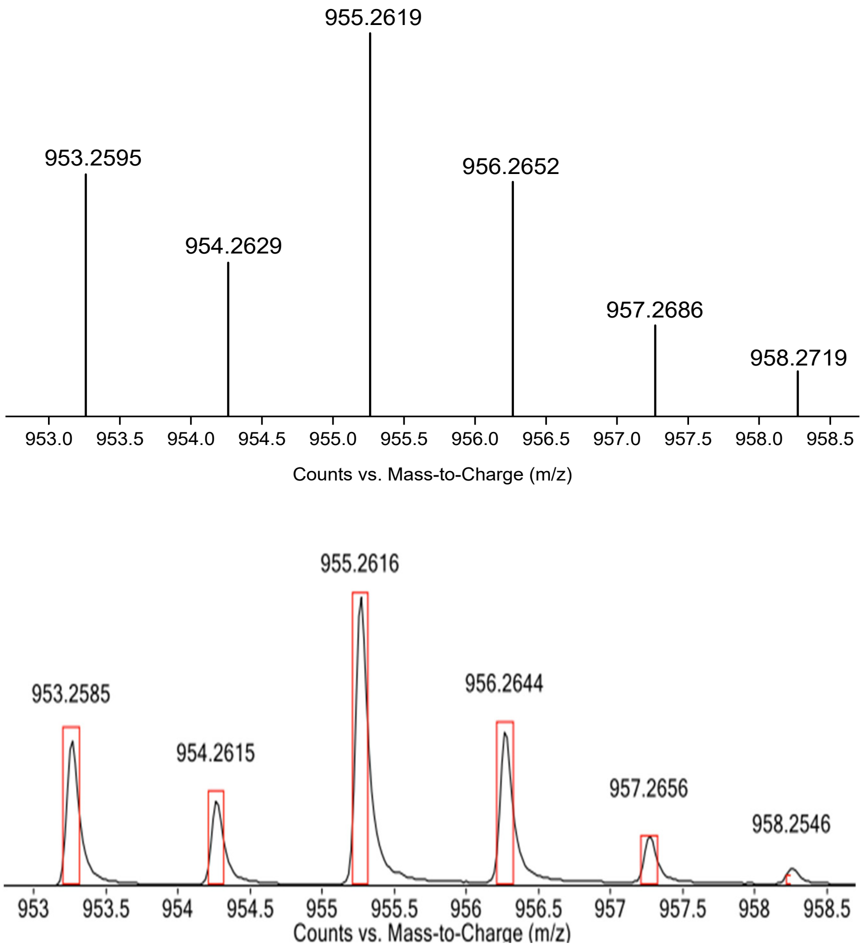

Fig. S20. Simulated (top) and experimental (bottom) ESI-MS data for complex **3b**, showing the isotropic distribution pattern for the molecular ion peak ($[M - PF_6]^+$).

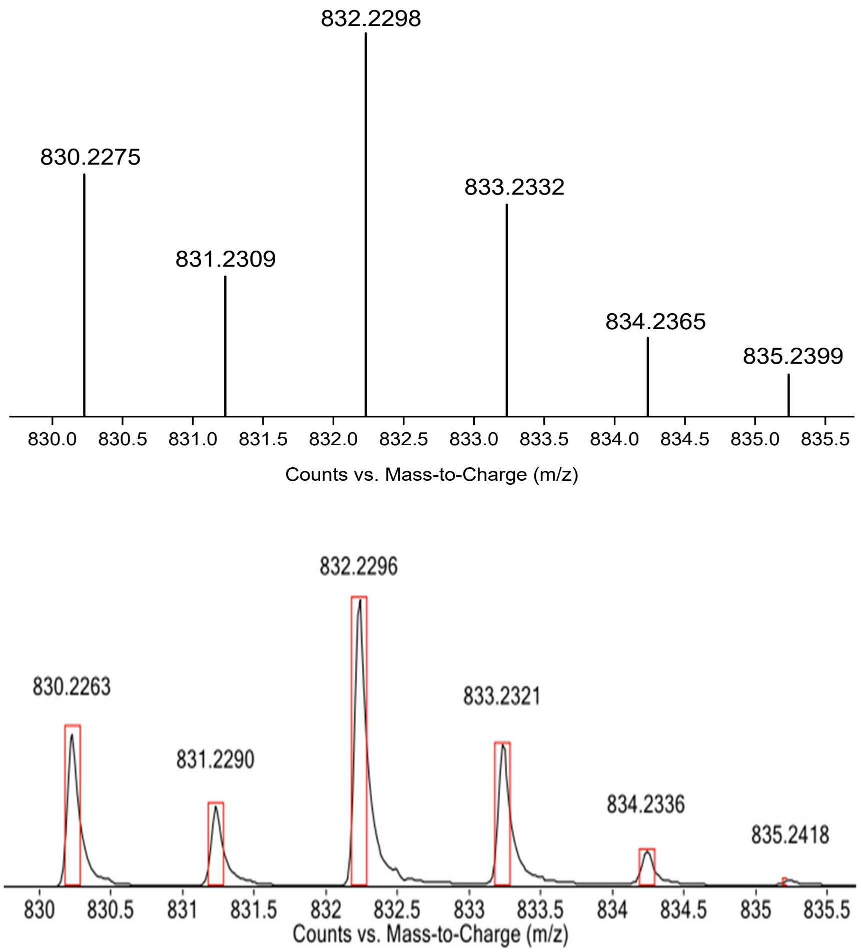

Fig. S21. Simulated (top) and experimental (bottom) ESI-MS data for complex **3c**, showing the isotropic distribution pattern for the peak ($[M - C_{14}H_9O_2NPF_6]^+$).

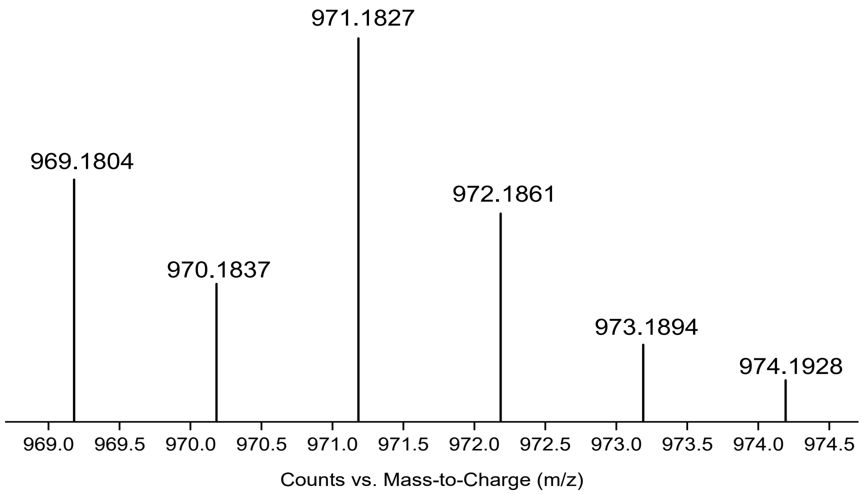

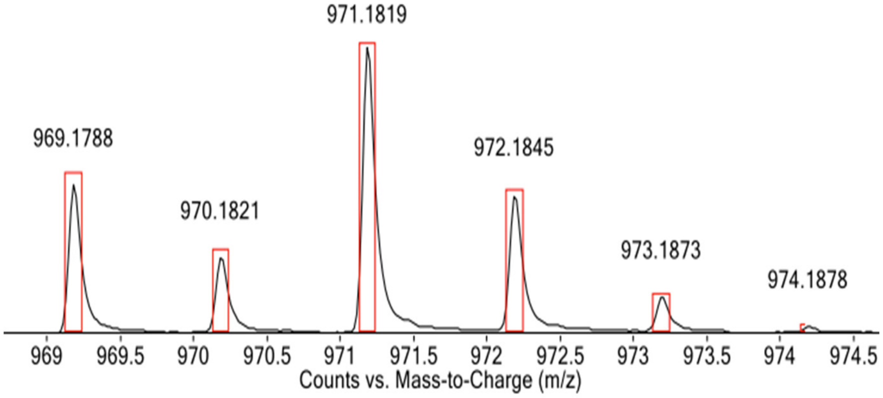

Fig. S22. Simulated (top) and experimental (bottom) ESI-MS data for complex **4a**, showing the isotropic distribution pattern for the molecular ion peak ($[M - PF_6]^+$).

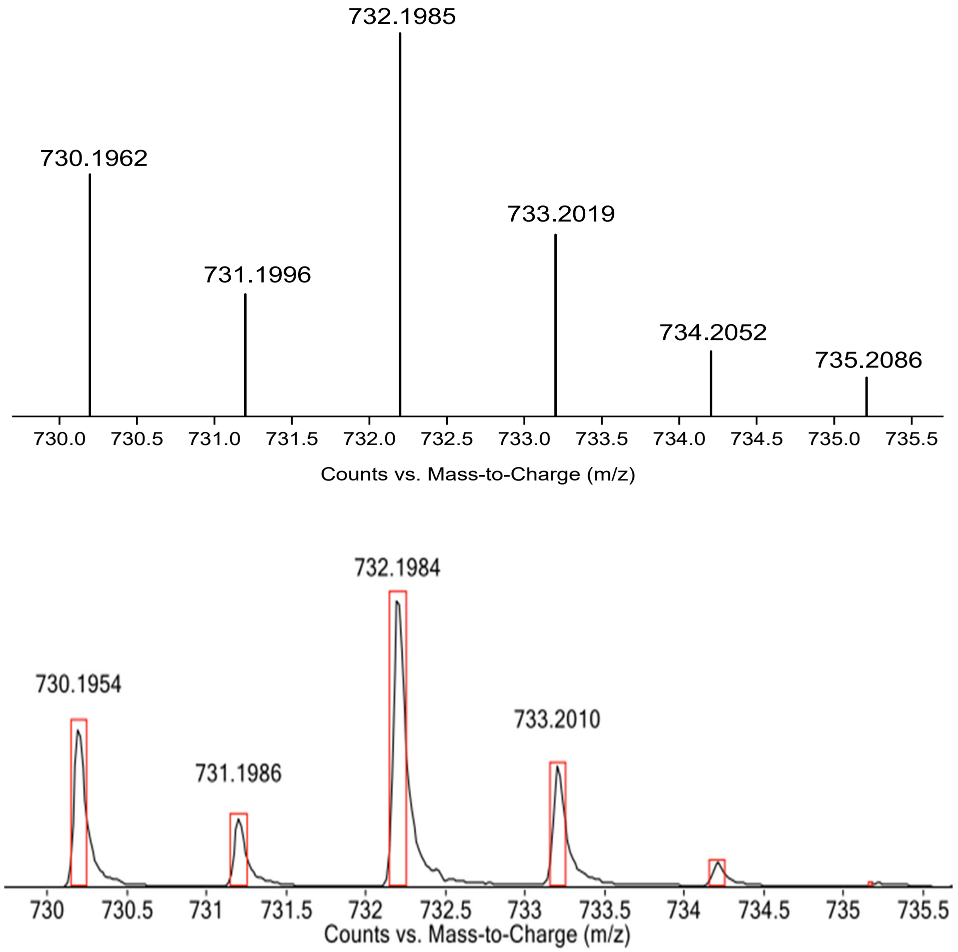

Fig. S23. Simulated (top) and experimental (bottom) ESI-MS data for complex **4b**, showing the isotropic distribution pattern for the peak ([M – $C_{15}H_9O_4NPF_6$]$^+$).

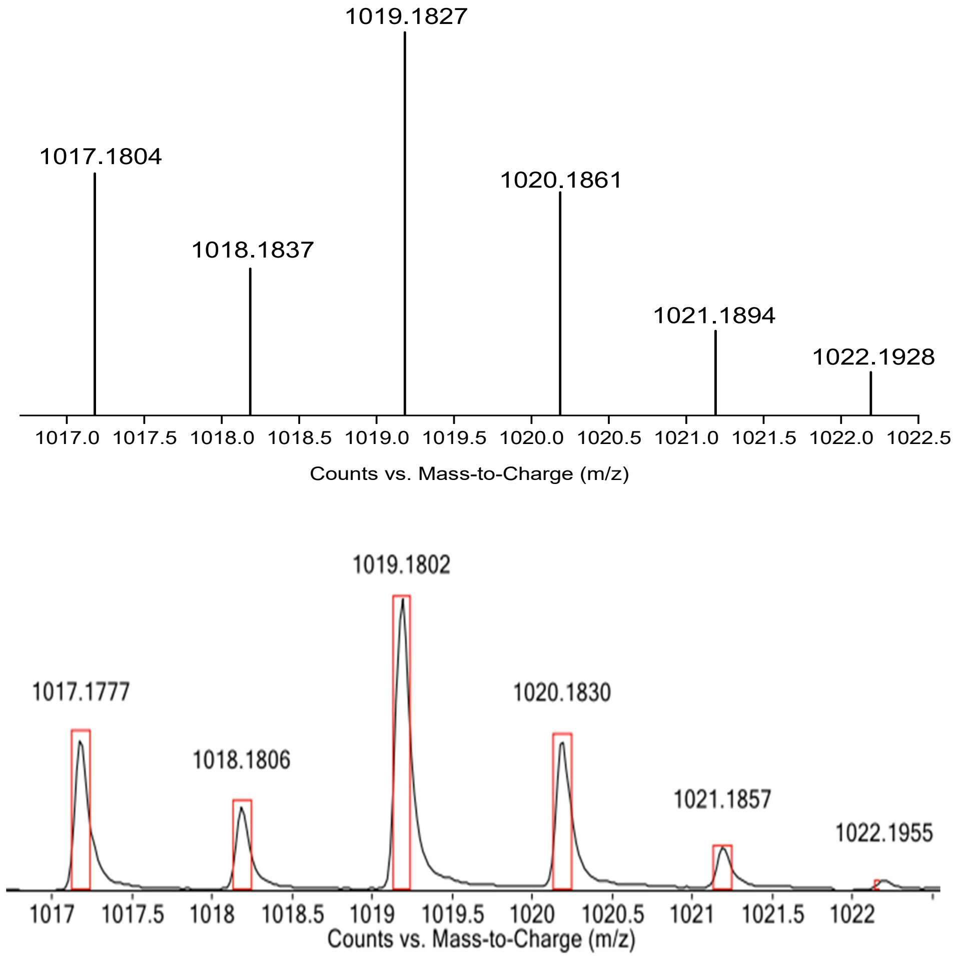

Fig. S24. Simulated (top) and experimental (bottom) ESI-MS data for complex **5a**, showing the isotropic distribution pattern for the molecular ion peak ($[M - PF_6]^+$).

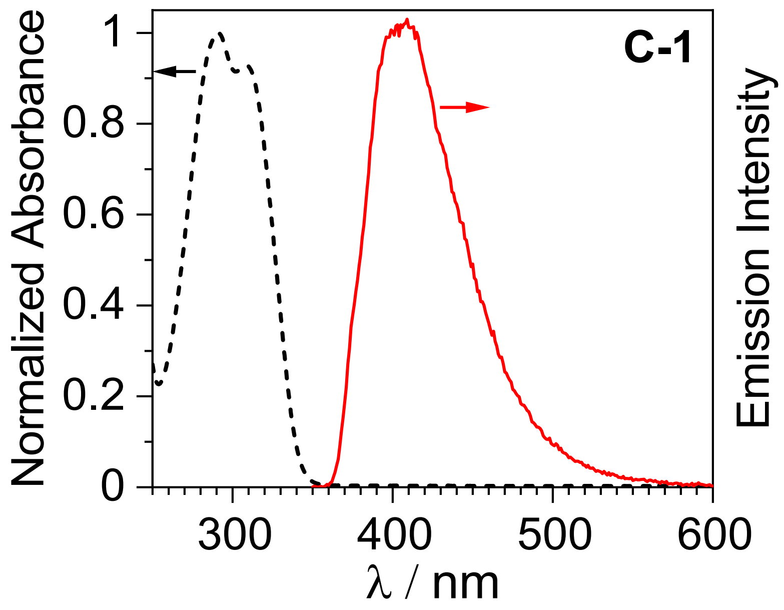

Fig. S25. Overlaid UV-vis absorption (black dashed line) and photoluminescence (red solid line) spectra of coumarin **C-1**, recorded at 293 K in CH_2Cl_2.

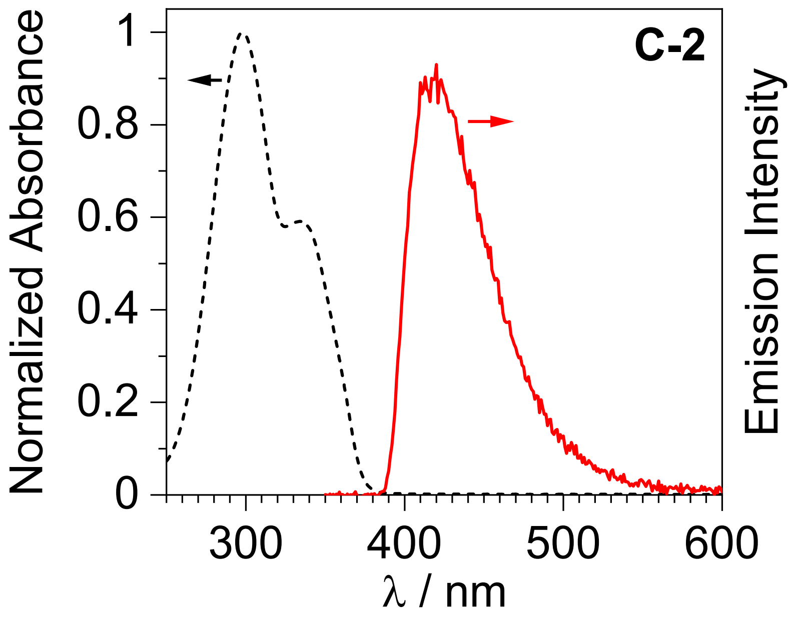

Fig. S26. Overlaid UV-vis absorption (black dashed line) and photoluminescence (red solid line) spectra of coumarin **C-2**, recorded at 293 K in CH_2Cl_2.

Table S2. Summary of photoluminescence data recorded at 77 K in a mixture of CH_2Cl_2/toluene (1:3 v/v).

	λ_{em}/nm (77 K)
**3a**	396, 443, 467, 525, 567 (max), 613
**3b**	576, 624 (max), 677
**3c**	406, 614, 655 (max)
**4a**	407, 443, 489, 516 (max), 556
**4b**	577, 625 (max), 679
**5a**	406, 444, 475, 521, 565 (max), 614

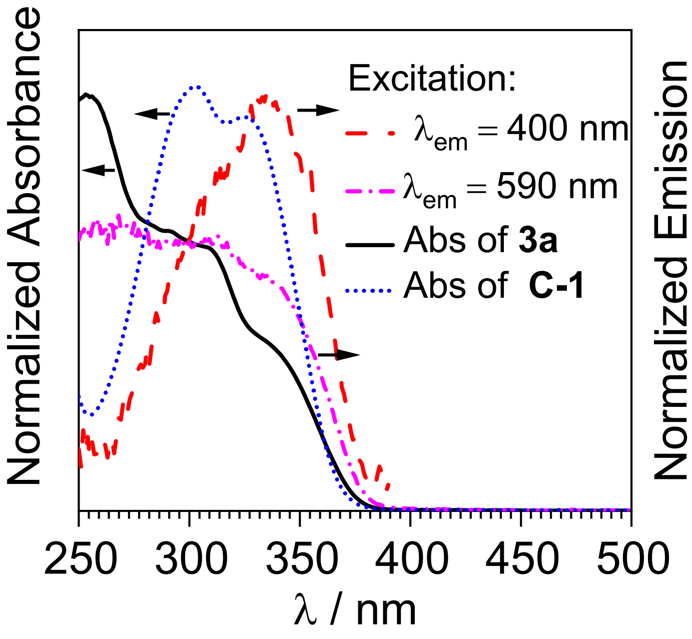

Fig. S27. Overlaid UV-vis absorption (black solid line) and excitation spectra of **3a**. Excitation spectra were monitored at the peak fluorescence (red dashed line) and phosphorescence (magenta dash-dot line) wavelengths. The UV-vis absorption spectrum of **C-1** (blue dotted line) is included for reference.

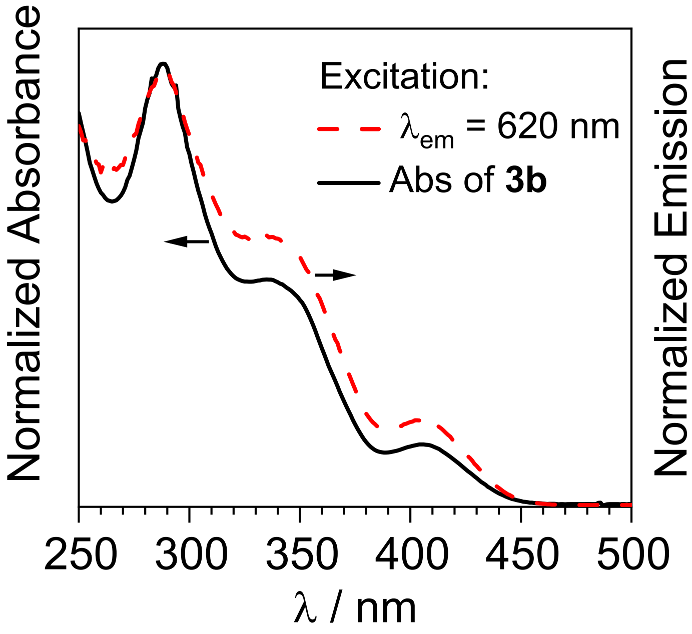

Fig. S28. Overlaid UV-vis absorption (black solid line) and excitation (red dashed line) spectra of **3b**. The excitation spectrum was monitored at the peak phosphorescence wavelength.

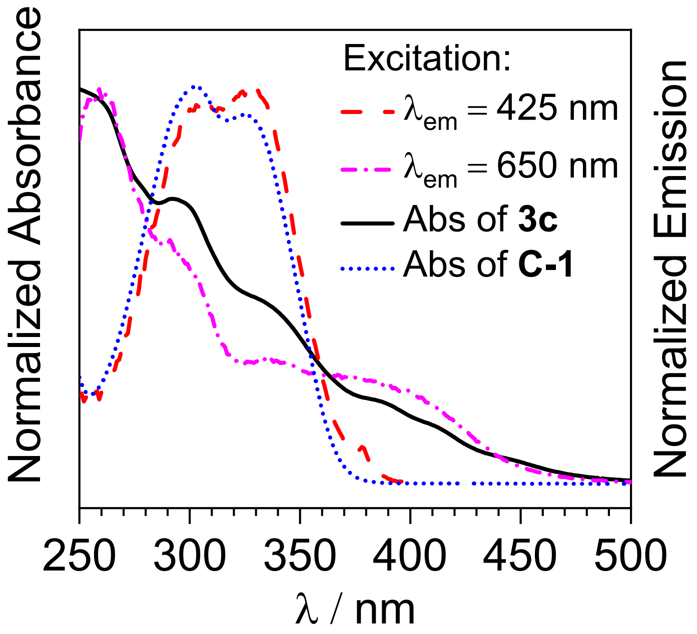

Fig. S29. Overlaid UV-vis absorption (black solid line) and excitation spectra of **3c**. Excitation spectra were monitored at the peak fluorescence (red dashed line) and phosphorescence (magenta dash-dot line) wavelengths. The UV-vis absorption spectrum of **C-1** (blue dotted line) is included for reference.

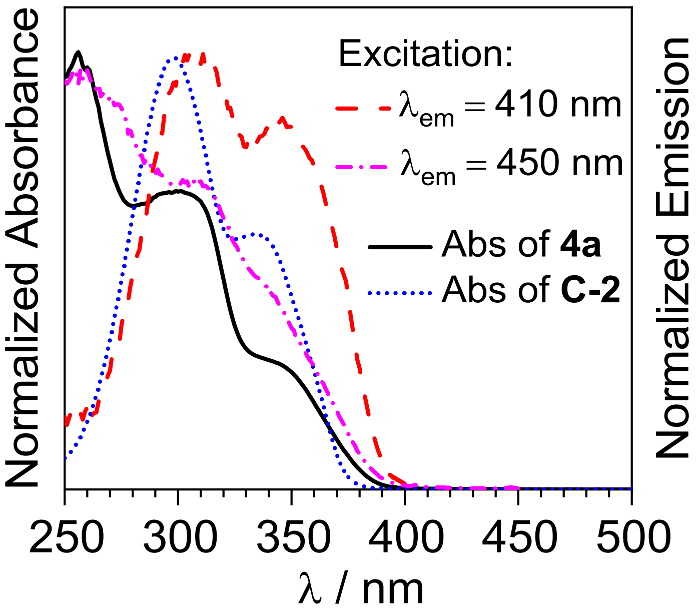

Fig. S30. Overlaid UV-vis absorption (black solid line) and excitation spectra of **4a**. Excitation spectra were monitored at the peak fluorescence (red dashed line) and phosphorescence (magenta dash-dot line) wavelengths. The UV-vis absorption spectrum of **C-2** (blue dotted line) is included for reference.

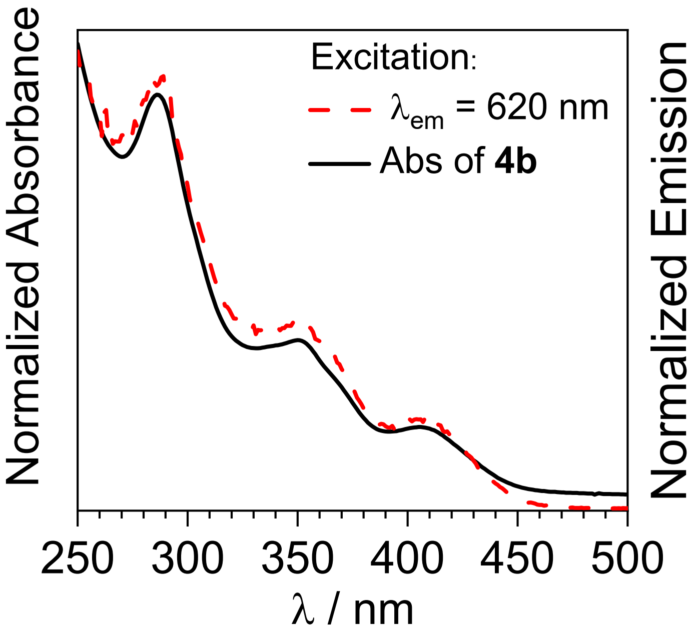

Fig. S31. Overlaid UV-vis absorption (black solid line) and excitation (red dashed line) spectra of 4**b**. The excitation spectrum was monitored at the peak phosphorescence wavelength.

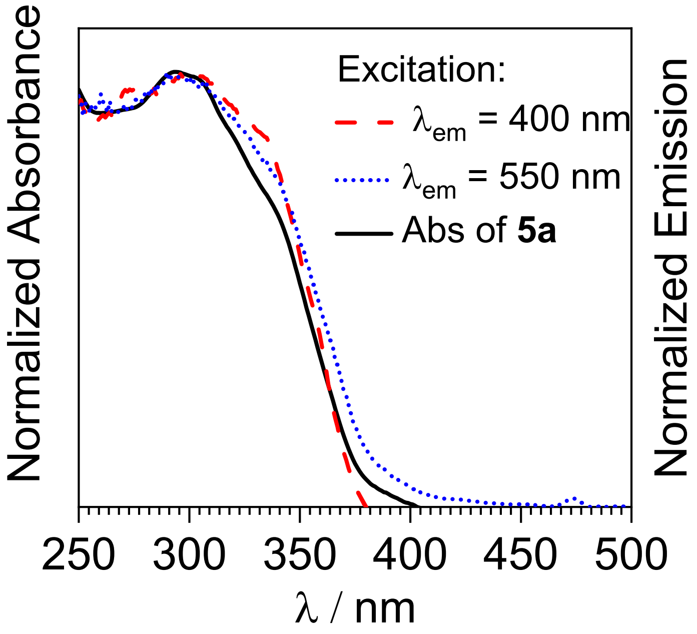

Fig. S32. Overlaid UV-vis absorption (black solid line) and excitation spectra of **5a**. Excitation spectra were monitored at the peak fluorescence (red dashed line) and phosphorescence (blue dotted line) wavelengths.

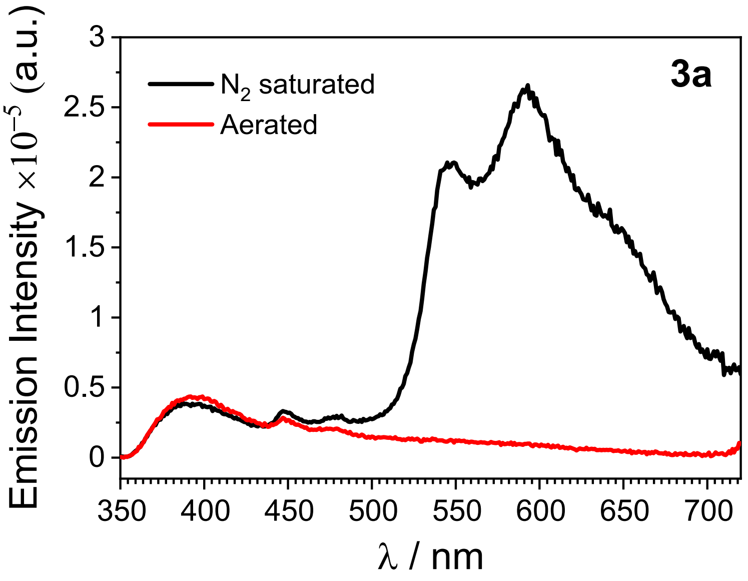

Fig. S33. Photoluminescence spectra of complex **3a** in deaerated and aerated solutions. Spectra were recorded at 293 K in CH_2Cl_2.

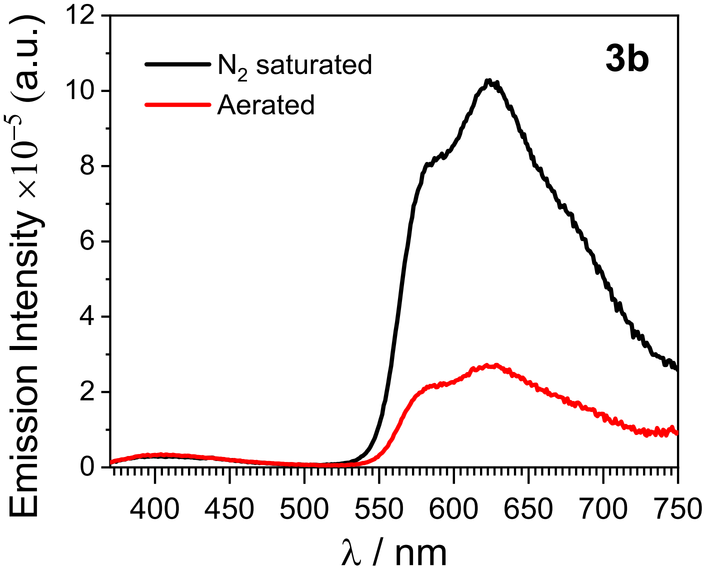

Fig. S34. Photoluminescence spectra of complex **3b** in deaerated and aerated solutions. Spectra were recorded at 293 K in CH_2Cl_2.

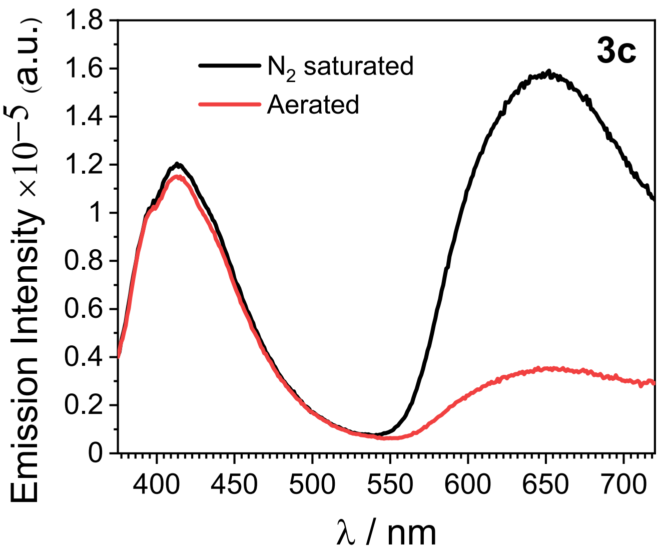

Fig. S35. Photoluminescence spectra of complex **3c** in deaerated and aerated solutions. Spectra were recorded at 293 K in CH_2Cl_2.

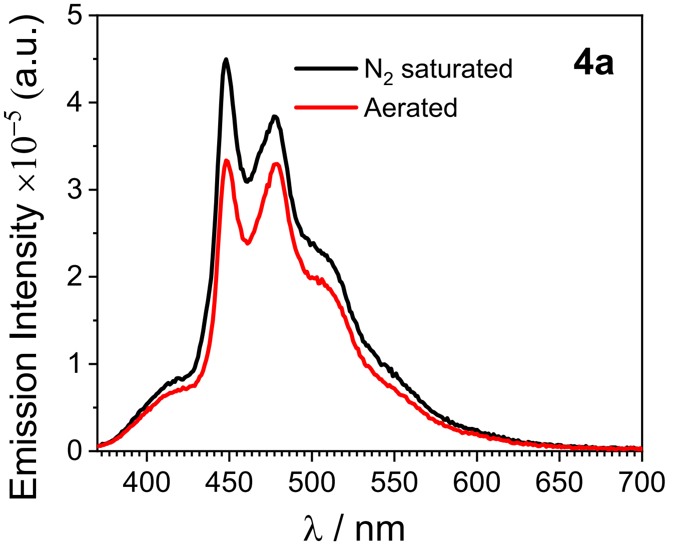

Fig. S36. Photoluminescence spectra of complex **4a** in deaerated and aerated solutions. Spectra were recorded at 293 K in CH_2Cl_2.

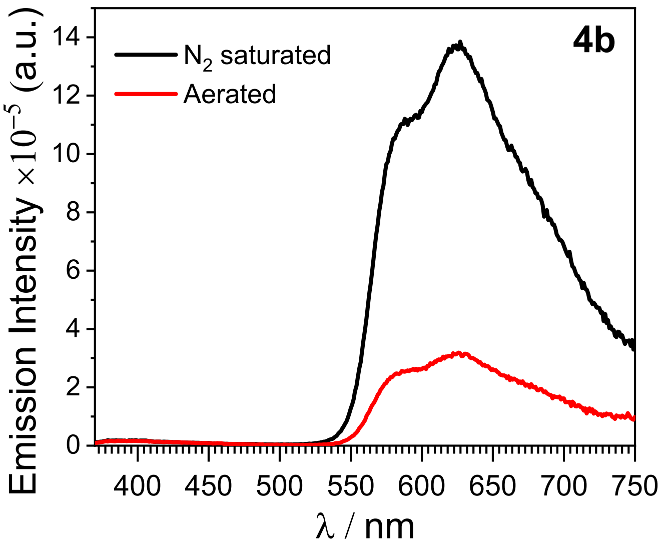

Fig. S37. Photoluminescence spectra of complex **4b** in deaerated and aerated solutions. Spectra were recorded at 293 K in CH_2Cl_2.

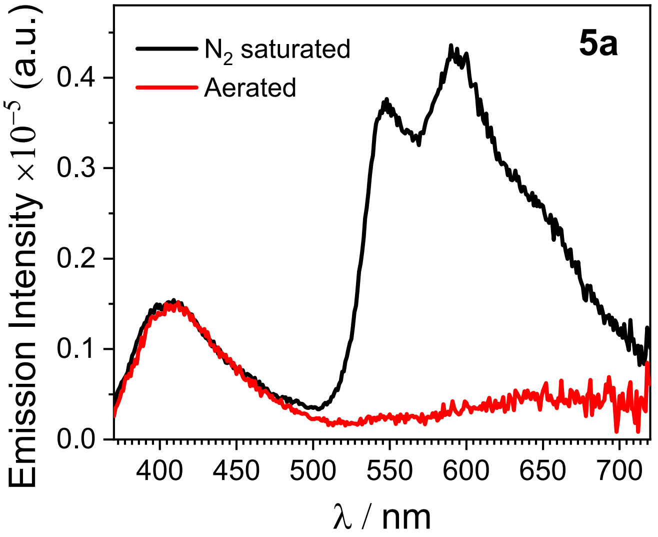

Fig. S38. Photoluminescence spectra of complex **5a** in deaerated and aerated solutions. Spectra were recorded at 293 K in CH_2Cl_2.

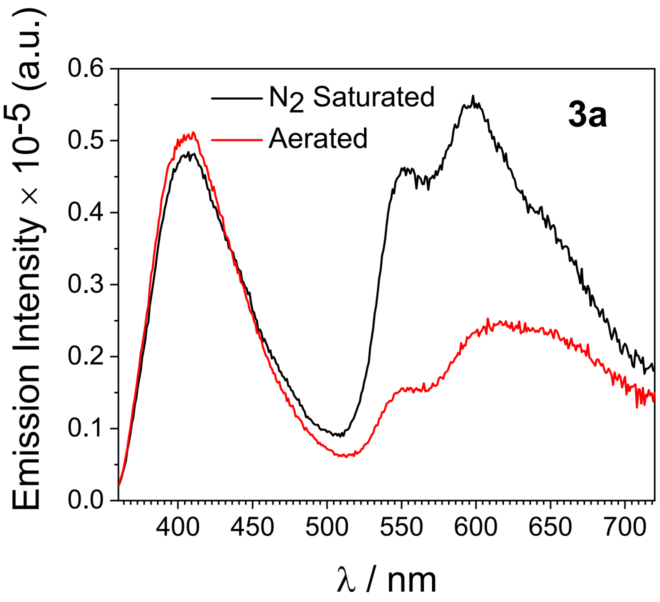

Fig. S39. Photoluminescence spectra of complex **3a** in deaerated and aerated solutions. Spectra were recorded at 293 K in deionized H_2O.

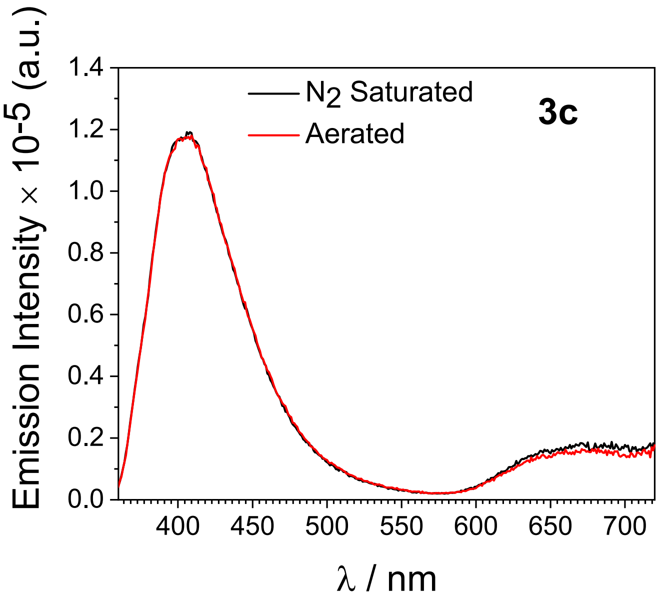

Fig. S40. Photoluminescence spectra of complex **3c** in deaerated and aerated solutions. Spectra were recorded at 293 K in deionized H_2O.

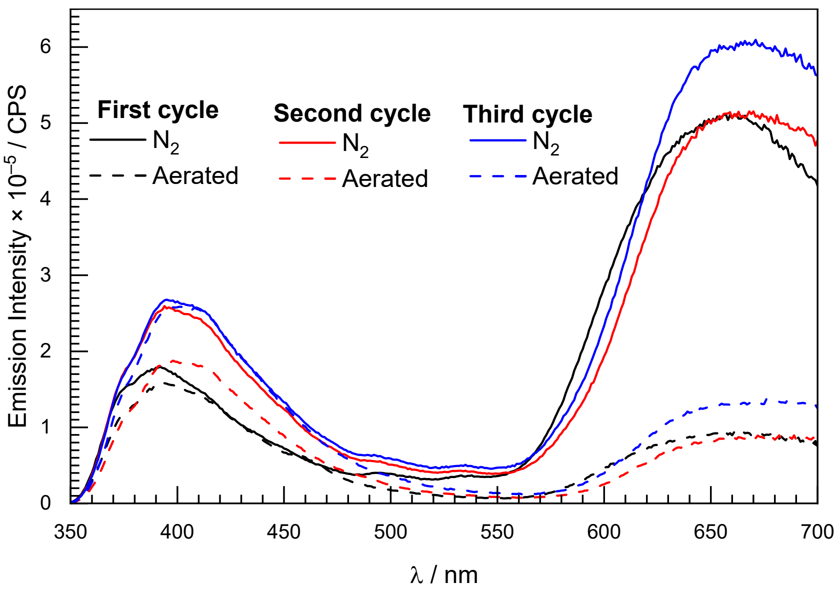

Fig. S41. Photoluminescence spectra of **3c** with repeated cycling of N_2 and aerobic atmospheres. 1) The initial N_2-saturated sample was prepared in a nitrogen-filled glovebox using deoxygenated CH_2Cl_2. 2) The sample was then exposed to air to obtain the aerated spectrum. 3) The aerated sample was deareated with three freeze-pump-thaw cycles on a Schlenk line, and then added back into the cuvette in the glovebox. This process was repeated over a total of three cycles.

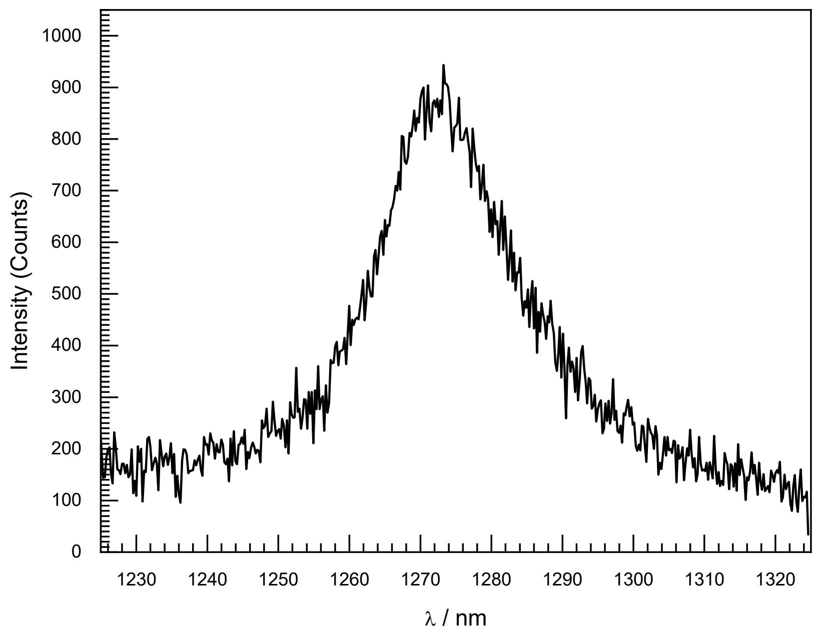

Fig. S42. Singlet oxygen emission spectrum upon irradiation of a solution $[Ru(bpy)_3](PF_6)_2$ in aerated DMF with 365 nm light. The absorbance of the solution at the excitation wavelength is 0.3.

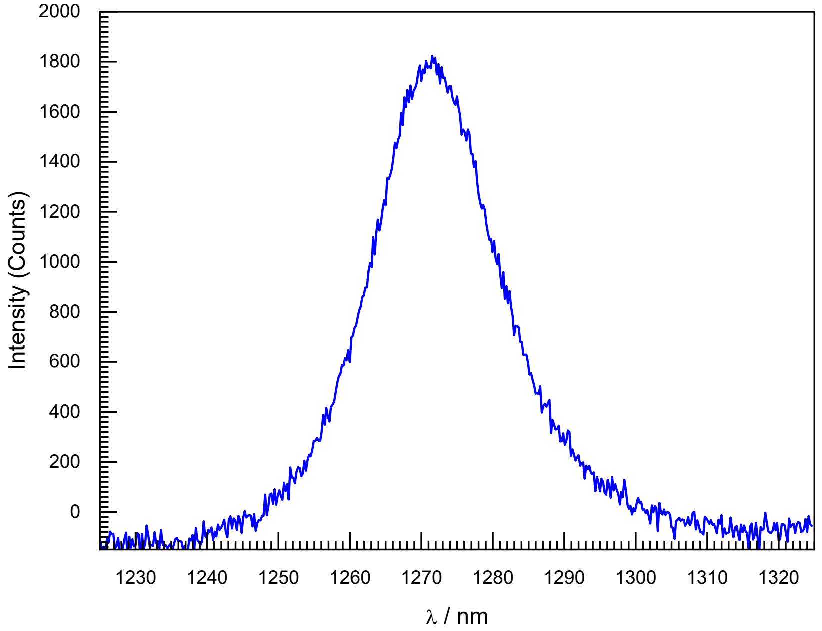

Fig. S43. Singlet oxygen emission spectrum upon irradiation of a solution **3a** in aerated CH_2Cl_2 with 365 nm light. The absorbance of the solution at the excitation wavelength is 0.3.

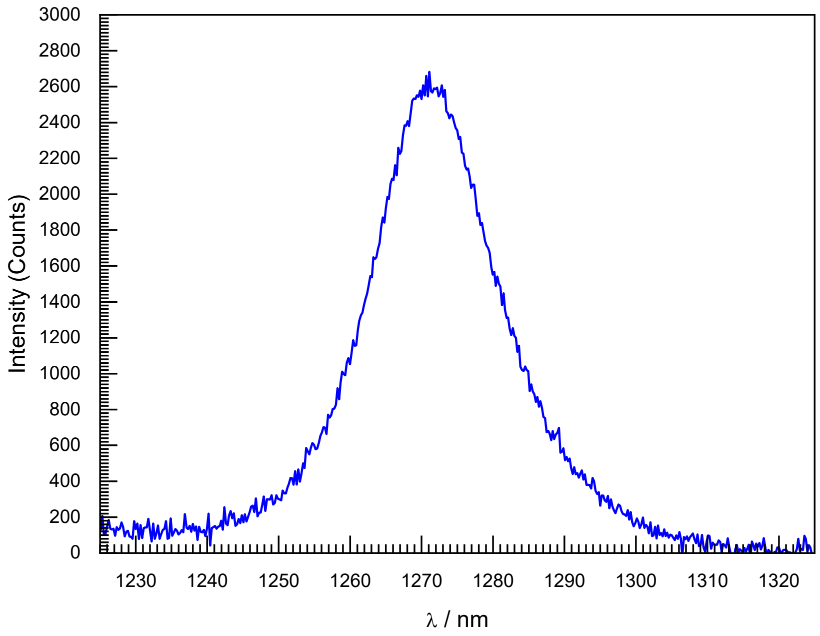

Fig. S44. Singlet oxygen emission spectrum upon irradiation of a solution **3c** in aerated CH_2Cl_2 with 365 nm light. The absorbance of the solution at the excitation wavelength is 0.3.

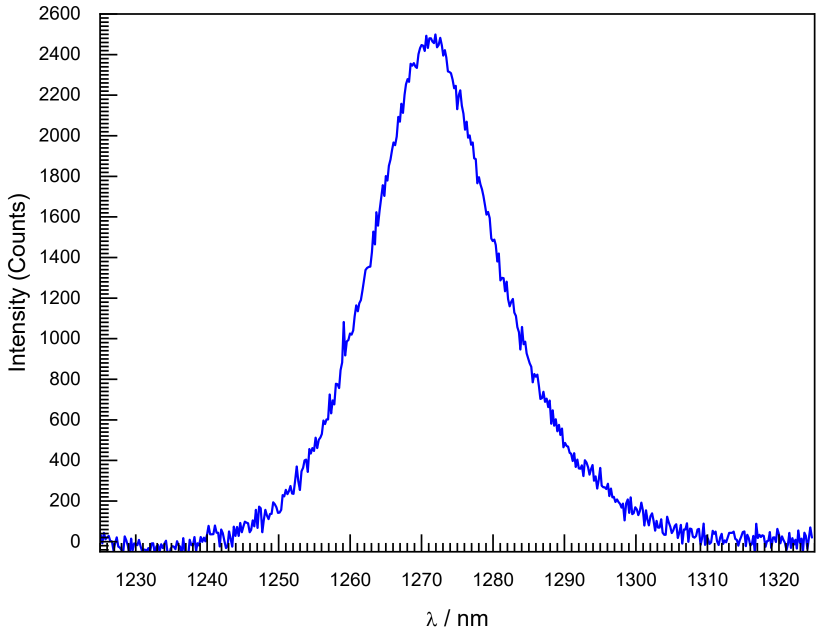

Fig. S45. Singlet oxygen emission spectrum upon irradiation of a solution **5a** in aerated CH_2Cl_2 with 365 nm light. The absorbance of the solution at the excitation wavelength is 0.3.

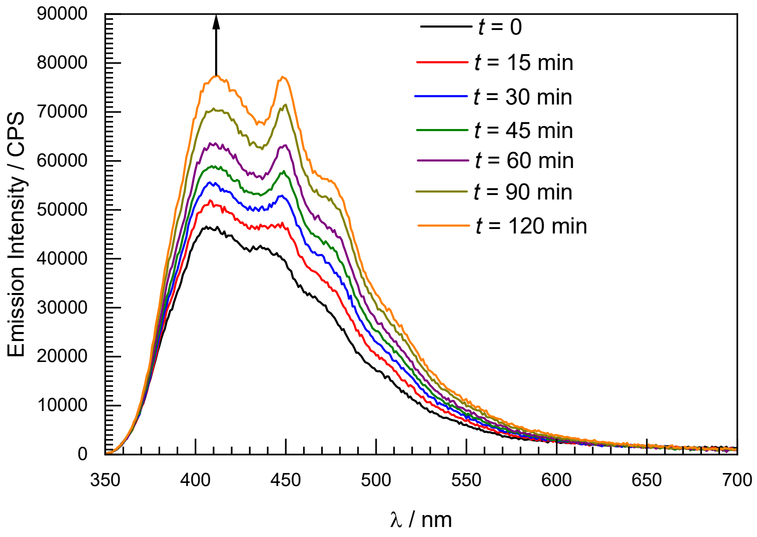

Fig. S46. Photoluminescence spectra of complex **3a** upon irradiation with 310 nm light, in CH_2Cl_2 under aerobic conditions.

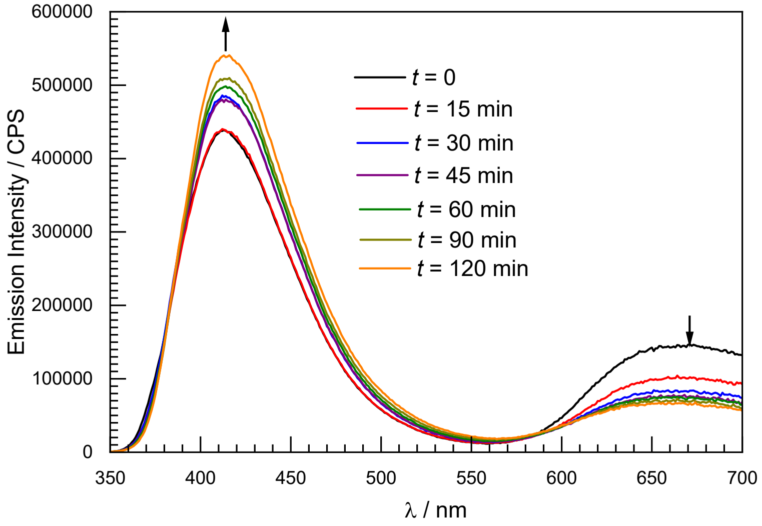

Fig. S47. Photoluminescence spectra of complex **3c** upon irradiation with blue light, in CH_2Cl_2 under aerobic conditions.

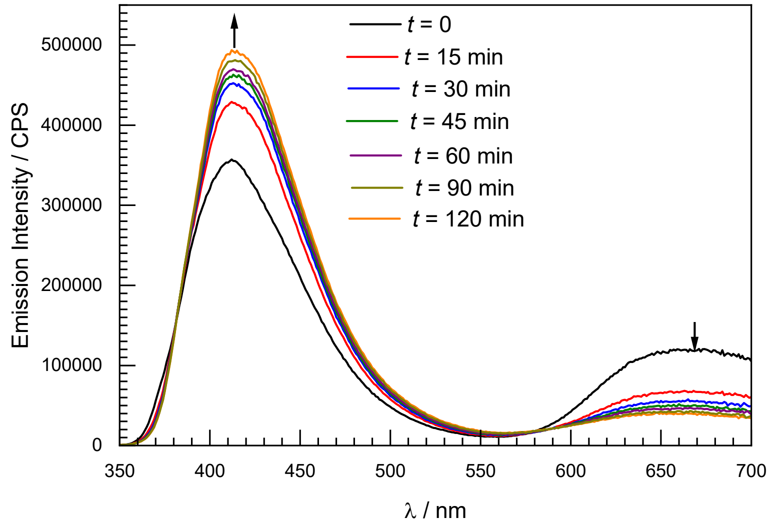

Fig. S48. Photoluminescence spectra of complex **3c** upon irradiation with 310 nm light, in CH_2Cl_2 under aerobic conditions.

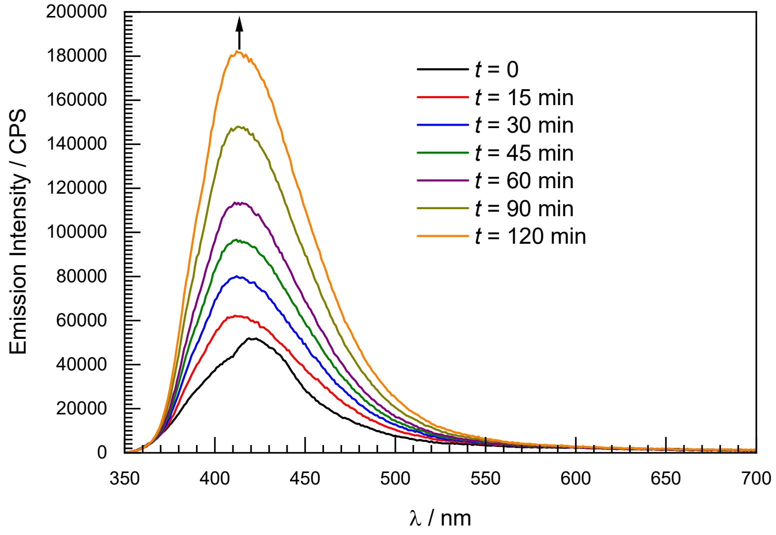

Fig. S49. Photoluminescence spectra of complex **5a** upon irradiation with 310 nm light, in CH_2Cl_2 under aerobic conditions.
